# Positive Feedback Keeps Duration of Mitosis Temporally Insulated from Upstream Cell-Cycle Events

**DOI:** 10.1016/j.molcel.2016.09.018

**Published:** 2016-10-20

**Authors:** Ana Rita Araujo, Lendert Gelens, Rahuman S.M. Sheriff, Silvia D.M. Santos

**Affiliations:** 1 Quantitative Cell Biology Lab, MRC-Clinical Sciences Centre (CSC), London W12 0NN, UK; 2 Institute of Clinical Sciences (ICS), Faculty of Medicine, Imperial College London, London SW7 2AZ, UK; 3 Laboratory of Dynamics in Biological Systems, Department of Cellular and Molecular Medicine, University of Leuven, 3000 Leuven, Belgium; 4 European Bioinformatics Institute, EMBL-EBI, Hinxton, Cambridge CB10 1SD, UK

**Keywords:** mitosis, positive feedback, temporal control, cell division, quantitative biology

## Abstract

Cell division is characterized by a sequence of events by which a cell gives rise to two daughter cells. Quantitative measurements of cell-cycle dynamics in single cells showed that despite variability in G1-, S-, and G2 phases, duration of mitosis is short and remarkably constant. Surprisingly, there is no correlation between cell-cycle length and mitotic duration, suggesting that mitosis is temporally insulated from variability in earlier cell-cycle phases. By combining live cell imaging and computational modeling, we showed that positive feedback is the molecular mechanism underlying the temporal insulation of mitosis. Perturbing positive feedback gave rise to a sluggish, variable entry and progression through mitosis and uncoupled duration of mitosis from variability in cell cycle length. We show that positive feedback is important to keep mitosis short, constant, and temporally insulated and anticipate it might be a commonly used regulatory strategy to create modularity in other biological systems.

## Highlights

• Duration of mitosis in single cells is short and remarkably constant
• Duration of mitosis is temporally insulated from variability in early cell-cycle phases
• Positive feedback keeps duration of mitosis short, constant, and insulated
• Positive feedback can be a cellular strategy to bring about modularity in signaling

## Graphical Abstract

**Figure:**
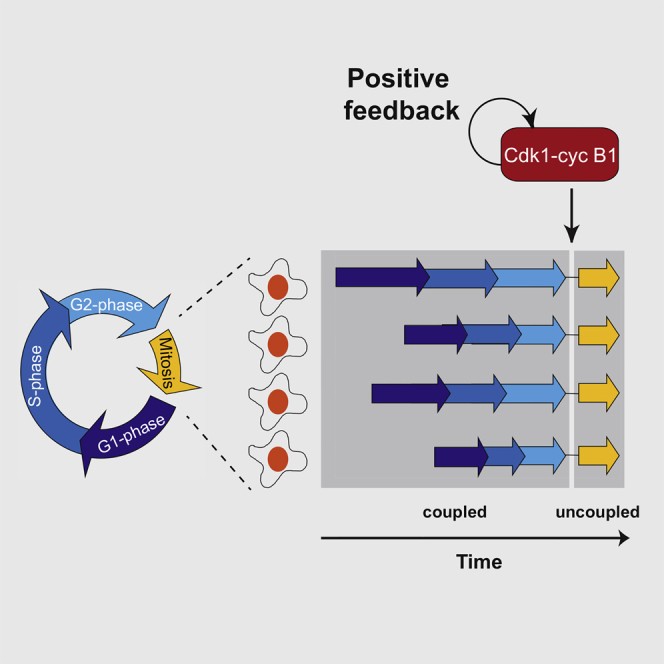

## Teaser

Araujo et al. show by quantitative single cell measurements that mitosis is short, fairly constant, and, surprisingly, uncoupled from the highly variable duration of earlier cell-cycle phases. They propose that positive feedback is responsible for this modularity and predict that it might enable modularity in other biological systems.

## Introduction

The cell cycle is characterized by a sequence of events by which a cell gives rise to two genetically identical daughter cells. In order to maintain the integrity of the genome, chromosome replication (S-phase) and chromosome segregation into the two daughter cells (M-phase) must be coordinated in a manner that ensures the faithful transmission of hereditary information from one generation of cells to the next. Indeed, the timely execution of each stage of the cell cycle is intimately linked to key developmental processes such as differentiation and organogenesis. On the other hand, failure to precisely regulate cell-cycle progression leads to various disease states such as cancer.

Transitions within the cell cycle have been thought to be regulated by activation and deactivation cycles of cyclin-dependent kinases (Cdks) (Solomon et al., 1990; and reviewed in Uhlmann et al., 2011) and of Cdk counteracting phosphatases (Bouchoux and Uhlmann, 2011), synthesis and degradation of regulatory cyclins (Murray et al., 1989, Murray and Kirschner, 1989), and activation and deactivation cycles of checkpoints (Hartwell and Weinert, 1989) at specific cell-cycle stages. These regulate proper ordering of cell-cycle phases and full completion of one phase before the onset of the next. The existence of such well conserved regulatory mechanisms and biochemical machinery that drives cell division in all animal cells would predict that cell division cycles might be similar in different cells. However, both within the same organism different cell types have different lengths of cell division cycles and within the same population cell-cycle length varies for individual cells (Minor and Smith, 1974).

**Figure 1:**
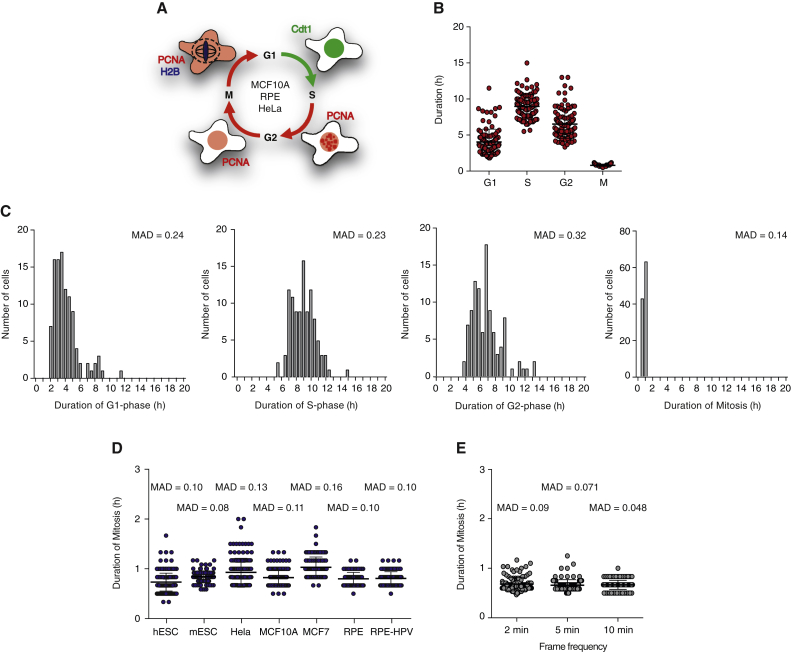
Duration of Mitosis Is Short and Constant (A) Schematic of cell lines and biosensors used to measure cell-cycle dynamics in single cells. (B) Duration of G1-, S-, G2-, and M-phases in single MCF10A cells. The error bars show mean ± SD (G1: 4.04 ± 1.78 hr, S: 8.97 ± 1.63 hr, G2: 6.52 ± 1.99 hr, and M: 48.0 ± 6.4 min). (C) Histograms showing duration of G1-, S-, G2-, and M-phases. The normalized MAD/mean are shown. n > 200 cells for each experimental condition. This is representative of n = 3 experiments. (D) Duration of mitosis in single cells as measured by live imaging for seven different human and mouse cell lines. H1 embryonic stem cells (hESC). R1 mouse embryonic cells (mESC). MAD is shown. (E) Duration of mitosis in single cells as measured by live imaging at different frame frequencies (2, 5, and 10 min per imaging frame). Mean ± SD (41.6 ± 8 min, 39 ± 6.6 min, and 40.2 ± 6 min for 2, 5, and 10 min, respectively) and MADs are shown. n > 100 cells were analyzed for each experimental condition.

**Figure 2:**
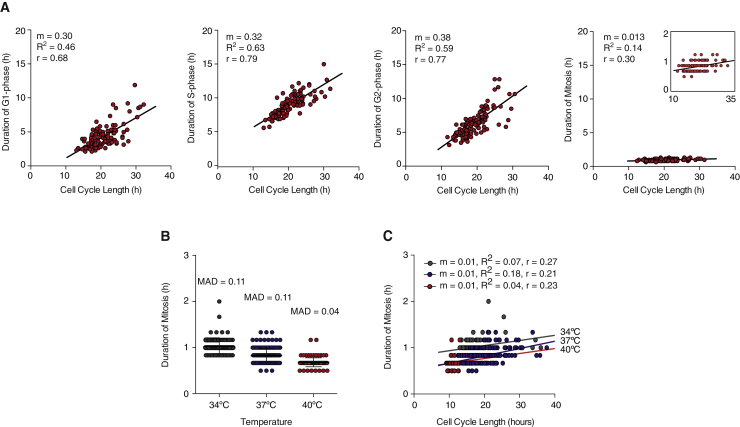
Duration of Mitosis Is Independent of Variability in Cell Cycle Length (A) Duration of G1-, S-, G2-, and M-cell-cycle phases in single cells as a function of cell-cycle length measured by single cell live imaging. The inset shows same data on a 0–2 hr y axis scale. (B) Duration of mitosis as measured by live cell imaging performed at 34°C, 37°C, and 40°C. The mean ± SD (61.6 ± 9.8 min, 49.4 ± 9.3 min, and 40.8 ± 5.8 min for 34°C, 37°C, and 40°C, respectively) and MAD are shown. (C) Duration of mitosis in single cells as a function of cell-cycle length measured at 34°C, 37°C, and 40°C. The trend lines with respective slope (m), R-squared (R^2^), and Pearso*n* r are shown. n > 100 cells were analyzed for each experimental condition.

Different models were devised to explain the temporal ordering of events during cell-cycle progression and, in particular, of mitosis: a threshold model (Lindqvist et al., 2007, Gavet and Pines, 2010) and a cascade model (Georgi et al., 2002). Whereas the former says that the various cell-cycle events require different threshold levels of Cdk activity, the latter would predict that early mitotic events promote the subsequent activation of the regulators of later mitotic events. Either model would predict that there should be a strong correlation between the duration of successive cell-cycle events. In other words, cells that are quick to complete one cell-cycle phase are more likely (than cells that did this phase slowly) to complete the next phase quickly. Here, we test whether this is true by combining quantitative measurements of cell-cycle dynamics using live cell imaging of cell-cycle biosensors and computational modeling. We find that, at the single cell level, the duration of G1-, S-, and G2-phases is highly variable and correlates well with cell-cycle length. However, the duration of mitosis is short, remarkably constant, and surprisingly uncoupled from variability in early cell-cycle phases (Figures 1 and 2). The question is thus, what controls proper duration of mitosis and ensures that it is a short fraction of the cell division cycle, uncoupled from timing of upstream events?

Entry and progression through mitosis depends on the activity of Cdk1 and its regulatory protein Cyclin B1. Work from many labs have described that Cdk1-cyclin B1 is embedded within positive and negative feedback regulation. The former relies on the ability of Cdk1-cyclin B1 to inhibit the activity of its own inhibitor, the kinase Wee1 (McGowan and Russell, 1995, Mueller et al., 1995, Tang et al., 1993) and activate its own activator, the phosphatase Cdc25 (Kumagai and Dunphy, 1992, Izumi et al., 1992). On the other hand, active Cdk1-cyclin B1 complexes activate the anaphase promoting complex APC-cdC20, which stimulates Cyclin B1 degradation and thereby Cdk1 inactivation, forming a negative feedback loop. It has been shown that these feedback loops allow Cdk1-cyclin B1 to have a switch-like activation and the Cdk1-cyclin B1 network to collectively function as a bistable trigger that helps make transition from interphase into mitosis all-or-none and irreversible in nature (Novak and Tyson, 1993, Sha et al., 2003, Pomerening et al., 2003).

This led us to hypothesize that positive feedback and bistability in the protein networks that regulate entry and progression through mitosis may result in the duration of mitosis remaining short, constant, and temporally insulated from temporal variability in earlier cell-cycle phases. Here, we test this hypothesis and find that, at the single cell level, and contrary to G1-, S-, and G2-phases, duration of mitosis is short, remarkably constant, and uncoupled from variability in cell-cycle duration. We show that checkpoint control alone cannot explain these properties and find that positive feedback in Cdk1-cyclin B1 regulatory network can account for the temporal insulation of mitosis. We show that compromising feedback control (both in the presence or absence of checkpoint activation) resulted in a sluggish mitotic entry and a slower, more variable progression into mitosis. Importantly, compromising positive feedback resulted in the coupling of duration of mitosis with cell-cycle length. In other words, a longer time completing G1-, S-, and/or G2-phase results in longer duration of mitosis. We therefore show that positive feedback can give rise to temporal insulation of mitosis. Finally, we formulate a simple theoretical model for entry and progression through mitosis, which accounts for the observed role of positive feedback as a control strategy to create modularity in cell-cycle regulation.

## Results

### Duration of Mitosis Is Short and Remarkably Constant

In order to measure cell-cycle dynamics in single cells, MCF10A (epithelial mammary) cells stably expressing Cdt1-YFP, PCNA-mCherry, and H2B-CFP fusions (Figures 1A and S1) were imaged for two consecutive divisions. G1 length was monitored by the appearance and disappearance of Cdt1 (Sakaue-Sawano et al., 2008). S-phase length was defined as the time between the appearance and disappearance of nuclear speckles (Sporbert et al., 2005). Duration of G2 was measured by monitoring time between disappearance of PCNA speckles and nuclear envelope breakdown (NEB). Duration of mitosis was defined by the time between NEB and nuclear envelope reformation (NER). Cell-cycle length was measured as the time between two consecutive NER events (Figures 1A and S1). The overall cell-cycle length of MCF10A cells is 21 hr long, on average (Figure 1B). Cells spend 95% of their cell division cycle in interphase (G1-, S-, and G2-phases) with average durations of 4 hr, 9 hr, and 5 hr to complete G1-, S-, and G2-phases, respectively. This results in cells spending only 5% of their cell-cycle time (less than 1 hr) in mitosis (Figures 1B–1D). Similar cell-cycle dynamics are seen for other human somatic cells such as RPE (epithelial, retina) and HeLa (epithelial, cervix) cells (Figure S1). In addition, measuring dynamics of individual cell-cycle phases revealed that mitosis is not only the shortest cell-cycle phase, but is also remarkably constant. Whereas timing of G1-, S-, and G2-phases gave rise to wide distributions with high (normalized) mean absolute deviations, MAD, and coefficients of variation, CV, (G1-phase: MAD = 0.24, CV = 0.44; S-phase: MAD = 0.23, CV = 0.28, and G2-phase: MAD = 0.32, CV = 0.30), the distribution of mitotic duration was tight, with little variability (normalized MAD = 0.14 and CV = 0.18) (Figure 1C). Similar results were seen for RPE and HeLa cells (Figure S1) and across seven human (and mouse) cell lines (Figure 1D), where the measured mitotic duration was on average 45 min (with MAD values between 0.08 and 0.16). Increasing the temporal resolution of acquired images to 2, 5, and 10 min showed no effect on duration of mitosis (average duration 42 min, with MAD < 0.09) (Figure 1E).

Altogether, these data suggest that despite variability in cell-cycle dynamics duration of mitosis is short and kept fairly constant in mammalian cells.

### Duration of Mitosis Is Temporally Insulated from Variability in Cell-Cycle Length

In addition, quantitative measurements of cell-cycle dynamics in MCF10A cells showed that in single cells, the relative cell-cycle length could be estimated by measuring duration of interphase. There is a good correlation between duration of individual cell-cycle phases and cell-cycle length (correlation coefficient, r, r = 0.68) (Figure 2A). In other words, if G1-, S-, or G2-phases are particularly long in a cell, the overall duration of the cell cycle for that cell is also long. Similar results were also seen for RPE and HeLa cells (Figure S2) and when correlating pairwise G1-, S-, and G2-phases (data not shown). However, this trend is not observed when duration of mitosis is analyzed in single cells. Regardless of the overall cell-cycle length for individual cells, there is very little correlation between duration of mitosis and cell-cycle length (r = 0.30) (Figure 2A). This was also observed for RPE and HeLa cells (Figure S2) and by monitoring cell-cycle dynamics in MCF10A, RPE, and HeLa cells with a different set of biosensors, DNA-ligase and Dnmt1 (Easwaran et al., 2005) and H2B (data not shown). This suggests that in single cells duration of mitosis is temporally uncoupled from variability in the duration of interphase.

To test this idea, we artificially slowed down and sped up the cell cycle by imaging MCF10A cells at low (34°C) and high (40°C) temperatures. Cells were monitored for two consecutive division cycles. Comparatively to control cells imaged at 37°C (where mitosis took on average 49.2 ± 5 min), duration of mitosis was longer (60 ± 9 min) at 34°C and shorter (40.8 ± 5.8 min) when cells were imaged at 40°C (Figure 2B). However, slowing or speeding up the cell cycle did not affect variability in mitotic duration, as measured by calculating CV (CV = 0.10 for 34°C, 37°C, and 40°C) and MAD values (MAD = 0.11 for 34°C; MAD = 0.11 for 37°C; and MAD = 0.04 for 40°C) (Figure 2B). This was also observed in RPE cells as shown in Figure S2. Notably, under these experimental conditions there was still very little correlation between duration of mitosis and overall cell-cycle length (Figures 2C and S2).

Altogether, these results suggest that duration of mitosis is temporally insulated (or uncoupled) from duration of upstream cell-cycle events.

### Perturbing Spindle Assembly Checkpoint Does Not Affect Temporal Insulation of Mitosis

**Figure 3:**
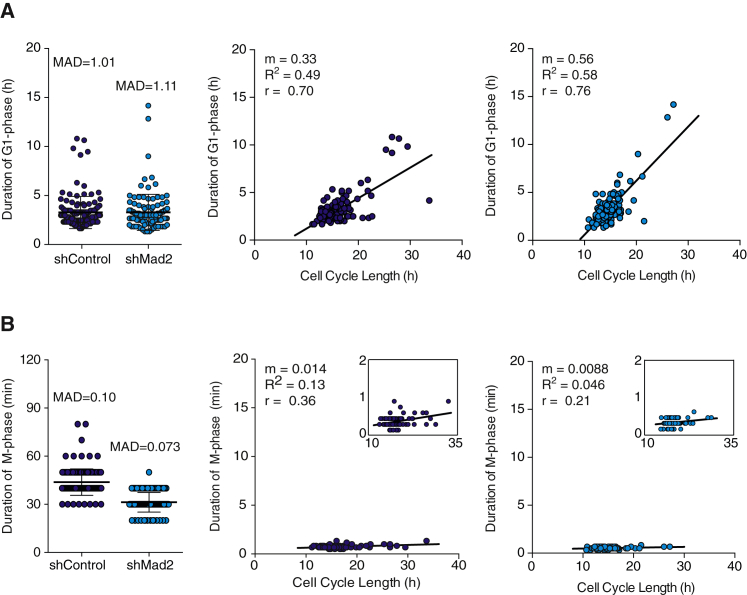
Perturbing the Spindle Assembly Does Not Make Mitotic Duration Variable nor Dependent on Cell Cycle Length (A) Left: duration of G1-phase measured in single cells in the presence (shControl) and absence (shMad2) of SAC. shScramble vector was used as control (shControl). The mean ± SD (shControl 3.30 ± 1.6 min and shMad2 3.28 ± 1.84 min) and MAD are shown. The duration of G1-phase in control cells plotted as a function of cell-cycle length is shown (middle). The duration of G1-phase in SAC perturbed (shMad2) cells plotted as a function of cell-cycle length is shown (right). The trend lines with respective slope (m), R-squared (R^2^), and Pearson r are shown. n > 110 cells were analyzed for each experimental condition. (B) Left: duration of mitosis measured in single cells in the presence (shControl) and absence of SAC (shMad2). shScramble vector was used as control (shControl). The mean ± SD (shControl 43.8 ± 8.2 min and shMad2 31.2 ± 6.1 min) and MADs are shown. The duration of mitosis in control cells plotted as a function of cell-cycle length is shown (middle). The duration of mitosis in SAC perturbed (shMad2) cells plotted as a function of cell-cycle length is shown (right). The trend lines with corresponding m and R^2^ are shown. Pearson r was calculated. The insets show same data on a 0–2 hr y axis scale. n > 115 cells were analyzed for each experimental condition.

We then set out to investigate what is the molecular mechanism underlying the observed modularity of mitosis. Transitions during the somatic cell cycle are heavily controlled by checkpoint regulation. Checkpoints assure that one cell-cycle phase begins only when the previous finishes and can thereby regulate the duration of individual cell-cycle phases (Hartwell and Weinert, 1989). The spindle assembly checkpoint (SAC) regulates metaphase to anaphase transition during mitosis (Gardner and Burke, 2000, Wells, 1996). SAC assures that segregation of chromosomes, progression to anaphase, and mitotic exit only happens once all the chromosomes are properly aligned in a metaphase plane and bound to microtubules, forming the mitotic spindle (reviewed in Wells, 1996, Gardner and Burke, 2000). Due to its key role in regulating duration of mitosis, it was conceivable that SAC could play a role in keeping duration of mitosis short, constant, and uncoupled from previous cell-cycle events. We tested this by downregulating the expression of Mad2, a crucial SAC component (Li and Murray, 1991), by 20%–40% with short hairpin RNA (shRNA) (Figure S3). MCF10A cells stably expressing either shMad2 or shScramble as control were imaged for two division cycles. In line with the prediction that Mad2 and SAC play a role specifically during mitosis, Mad2 downregulation had no effect on duration of G1 (shControl 3.3 ± 1.6 hr and shMad2 3.3 ± 1.8 hr, p value > 0.7 by Mann-Whitney test) (Figure 3A), but resulted in a shorter duration of mitosis (shControl 43.8 ± 8 min and shMad2 31.2 ± 6 min, p < 0.0001 Mann-Whitney test) (Figure 3B). This was also observed in RPE and HeLa cells as shown in Figure S3. However, even in SAC perturbed cells, duration of mitosis is kept constant (with low variability) when compared to control cells (shControl MAD = 0.10 and shMad2 MAD = 0.073) in the three cell lines tested (Figures 3B and S3). As seen previously, even in the absence of SAC, there is a high correlation between duration of G1-phase and cell-cycle length (shControl, r = 0.70 and shMad2, r = 0.76) (Figure 3A). Remarkably, perturbing Mad2 expression and SAC had no effect in insulating duration of mitosis from cell-cycle length, as seen by the lack of correlation between duration of mitosis and cell-cycle duration (shControl, r = 0.36 and shMad2, r = 0.21) (Figure 3B). Experiments performed in RPE and HeLa cells showed similar results (Figure S3).

Taken together, these results suggest that while SAC plays a role in regulating duration of mitosis, it is not the molecular mechanism that keeps duration of mitosis short, constant, and temporally insulated from upstream cell-cycle events.

### Positive Feedback Keeps Mitosis Temporally Insulated from Variability of Early Cell-Cycle Events

The question remained of what could the molecular mechanism be? Mitotic onset is initiated by a bistable trigger, which is thought to be the basis for a unidirectional, all-or-none and irreversible entry and progression through mitosis. (Novak and Tyson, 1993, Sha et al., 2003, Pomerening et al., 2003, Santos et al., 2012). A switch-like activation (Tyson and Novak, 2001, Thron, 1996, Sha et al., 2003, Pomerening et al., 2003) and spatial redistribution of Cdk1-cyclin B1 due to positive feedback control (Santos et al., 2012) at the onset of mitosis is at the heart of the observed bistability. Compromising the abruptness of Cdk1-cyclin B1 activation and nuclear import has been shown to affect progression through mitosis (Holt et al., 2008, Santos et al., 2012).

**Figure 4:**
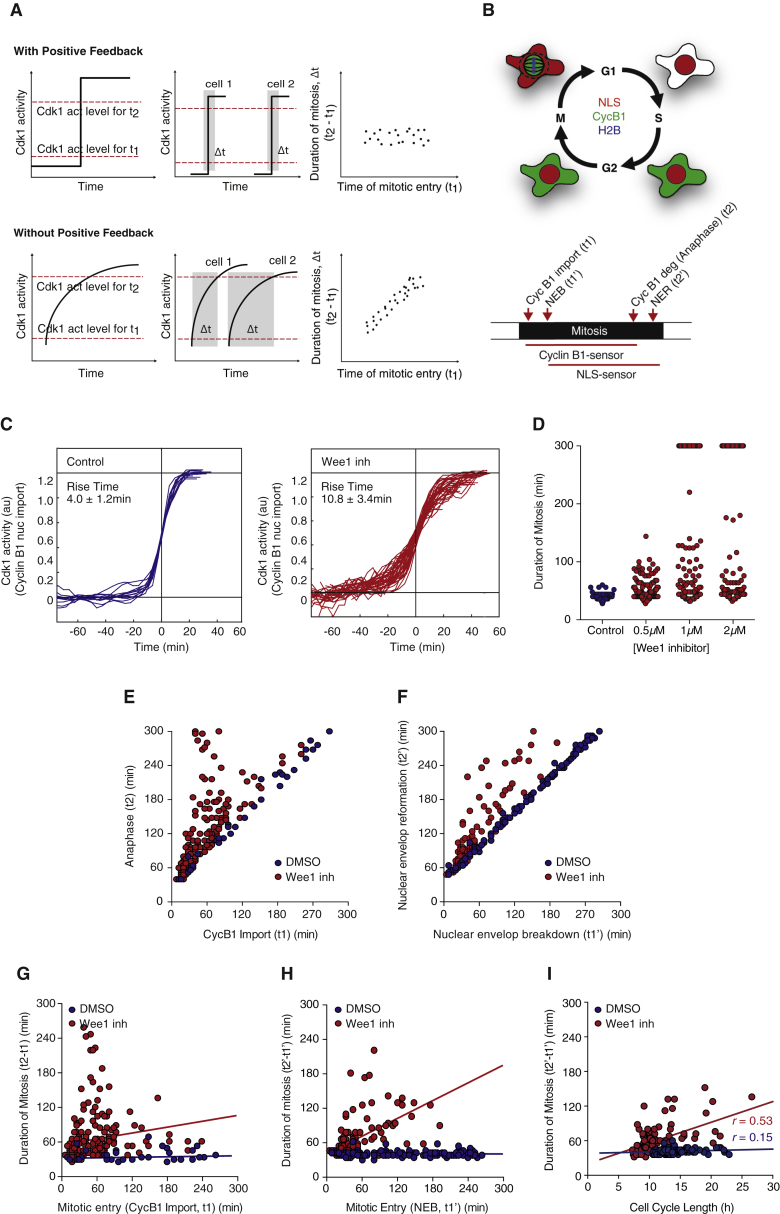
Positive Feedback Keeps Mitosis Temporally Insulated from Upstream Cell Cycle Events (A) Schematic of the thought experiment to test importance of positive feedback in keeping duration of mitosis constant and uncoupled from previous cell-cycle events. The presence of positive feedback results in a sharp, sigmoidal activation of Cdk1 and a short, constant time between an early (t1) and a late (t2) mitotic events (top). This may result in two cells entering mitosis at different times to keep a short and constant time between t1 and t2 (Δt). As a consequence, there might be no correlation between the time at which individual cells entered mitosis (t1) and duration of mitosis (t2-t1). The absence of positive feedback results in a graded, hyperbolic activation of Cdk1 and a long, variable time between an early (t1) and a late (t2) mitotic event (bottom). Consequently, there might be some degree of correlation between the time at which cells entered mitosis (t1) and duration of mitosis (t2-t1). (B) Schematic of stable cell lines and biosensors used to measure Cdk1 activity and early (Cyclin B1 import and nuclear envelope breakdown [NEB]) and late (Anaphase and nuclear envelope reformation [NER]) mitotic events. (C) Quantification of Cdk1 activation over time in single cells in the absence (blue) or presence (red) of 1 μM Wee1 inhibitor, PD166285. Time courses of individual cells were fitted to the logistic equation y = a+b/1+e^−(t−t0/τ)^ and were scaled to their fitted maximum and minimum values (b and a, respectively) and half-maximal times (t0). The rise times (τ) were calculated from the curve fits for all cells and are expressed as means ± SD. n > 20 cells in each condition. (D) Duration of mitosis in single cells in the presence (red) and absence (blue) of Wee1 inhibitor at the shown concentrations. n > 100 cells were analyzed for each experimental condition. (E) Time of anaphase as a function of Cyclin B1 nuclear import in cells either treated with DMSO (blue) or with Wee1 inhibitor (red). (F) Time of NER as a function of NEB in cells either treated with DMSO (blue) or with Wee1 inhibitor (red). n > 200 cells were analyzed for each experimental condition. (G) Duration of mitosis (measured by the time between Cyclin B1 nuclear import and the onset of anaphase) as a function of Cyclin B1 import (t1) in the presence (red) or absence (blue) of 1 μM Wee1 inhibitor. The trend lines are shown (blue line: m = 0.015, R^2^ = 0.012, r = 0.16 and red line: m = 0.17, R^2^ = 0.025, r = 0.16). (H) Duration of mitosis (measured by the time between NEB and NER) and the onset of NEB (t1’) in the presence (red) or absence (blue) of Wee1 inhibitor. DMSO was used as control. n > 200 cells were analyzed for each experimental condition. The trend lines are shown. (blue line: m = 0.0029, R^2^ = 0.0022, r = 0.047 and red line: m = 0.52, R^2^ = 0.30, r = 0.55). (I) Duration of mitosis (measured by the time between NEB and NER) as a function of cell-cycle length in the presence (red) or absence (blue) of 0.5 μM Wee1 inhibitor. The trend lines are shown (blue line: m = 0.004, R^2^ = 0.021, r = 0.15 and red line: m = 0.060, R^2^ = 0.28, r = 0.53). n > 100 cells were analyzed for each experimental condition.

We hypothesized that positive feedback could be the molecular mechanism underlying the short, constant duration of mitosis and its modular character. How could this work? Cdk1 activation is required for mitotic entry and progression into mitosis. As shown schematically in Figure 4A, it is plausible that early mitotic events (t1) require lower levels of Cdk1 activity, while late mitotic events (t2) require perhaps higher levels. The presence of positive feedback gives rise to a fast, sharp, sigmoidal activation of Cdk1. As a result, regardless of when individual cells enter mitosis, the time that it takes to go from an early to a late mitotic event (Δt) is likely to be short and relatively constant. Consequently, no correlation is expected between when individual cells initiate mitosis and duration of mitosis (Figure 4A). When positive feedback is compromised, however, Cdk1 switch-like activation is also compromised. We expect this to result in a more sluggish and more variable entry and progression through mitosis (Santos et al., 2012). Depending on how strongly positive feedback is compromised in individual cells, cells that initiate mitosis early might finish mitosis early as compared to cells that started mitosis later. This may result in a correlation (or coupling) between when individual cells entered mitosis and duration of mitosis (Figure 4A).

In order to test whether positive feedback could underlie temporal insulation in mitosis, Cdk1 activation dynamics as well as early and late mitotic events (i.e., duration of mitosis) were measured in the presence and absence of positive feedback in single cells. MCF10A cells stably expressing Cyclin B1-YFP, NLS-mCherry, and H2B-CFP biosensors were used (Figure 4B). Cyclin B1 nuclear translocation was used as a proxy for Cdk1 activation, since Cyclin B1 redistribution at the onset of mitosis is dependent on Cdk1 activity (Santos et al., 2012). Time of Cyclin B1 nuclear import, as well as time of NEB, were measured as early mitotic events (t1). Time of Cyclin B1 degradation, as well as time of NER, were measured as late mitotic events. Duration of mitosis was estimated as the time between Cyclin B1 import and its degradation and/or the time between NEB and NER (Figure 4B). Positive feedback was compromised by 75% by treating cells with the small molecule PD 166285, a specific Myt1/Wee1 inhibitor (Figure S4) (Hashimoto et al., 2006). Perturbing positive feedback resulted in a more graded Cdk1 activation, as seen by the increased rise time of Cdk1 activation curves (Figure 4C). Similar results were seen in RPE cells (Figure S4).

Notably, cells where positive feedback is compromised took longer to complete mitosis, presumably because it took longer for the cells to satisfy the spindle assembly checkpoint and/or activate APC-cdc20 (Figure 4D), and showed a more variable duration of mitosis (Figure 4D). In addition, breaking positive feedback resulted in loss of synchronicity between early (Cyclin B1 import and NEB) and late mitotic events (Cyclin B1 degradation and NER) (Figures 4E and 4F). Importantly, in control cells with intact feedback regulation, the length of mitosis is kept constant and there is no correlation (r = 0.047) between the time at which individual cells entered mitosis (t1 and t1′) and duration of mitosis (t2-t1 or t2′-t1′) (Figures 4G and 4H), emphasizing the independence of these events. Strikingly, when positive feedback is compromised, there is a correlation (r = 0.55) between the time at which cells entered mitosis and duration of mitosis (Figures 4G and 4H). Measuring early and late events with both Cyclin B1-YFP and NLS-mCherry biosensors provided similar results (Figures 4G and 4H). Similar results were also seen in both RPE and HeLa cells (Figure S4). Notably, measuring cell-cycle length between two consecutive divisions (time between NER of the first division and NEB of the second division) showed that perturbing positive feedback couples variability in cell-cycle length to duration of mitosis (Figures 4H and S4I).

**Figure 5:**
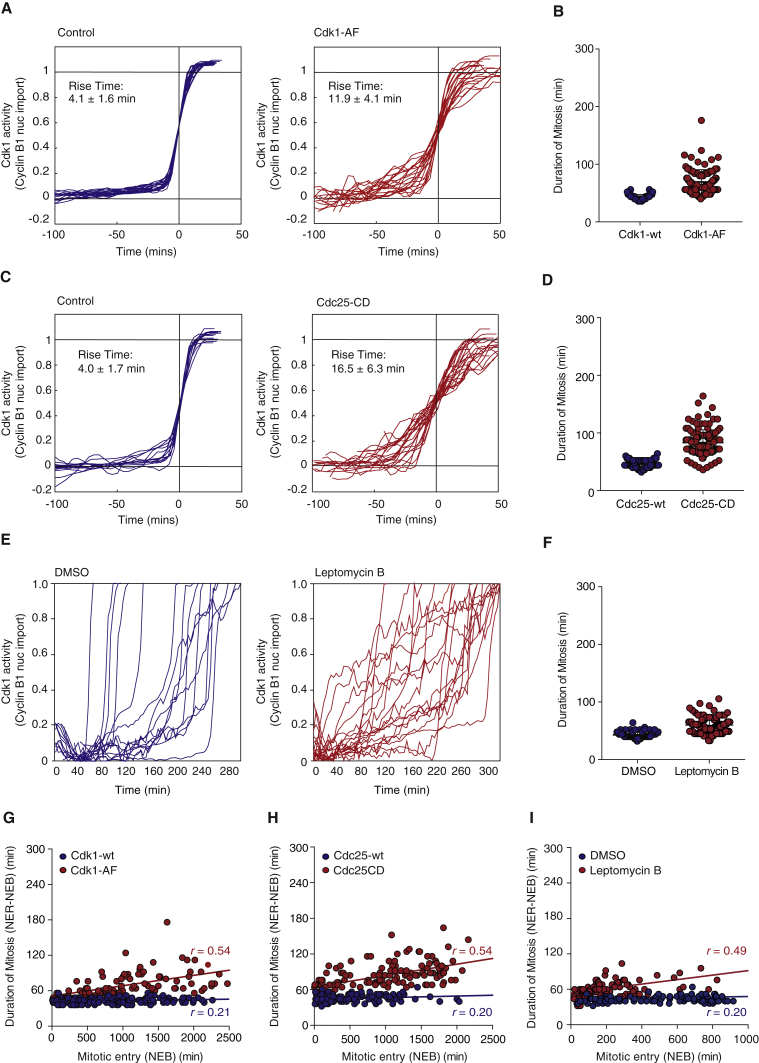
Breaking Cdk1 Activation and Spatial Positive Feedbacks Couples Duration of Mitosis to Upstream Cell-Cycle Events (A) Quantification of Cdk1 activation over time in cells expressing Cdk1-wt (blue) or Cdk1-AF (red). The time courses of individual cells were fitted as described in Figure 4C. Rise times (τ) were calculated from the curve fits for all cells and are expressed as means ± SD. n > 20 cells in each condition. (B) Duration of mitosis in cells ectopically expressing Cdk1-wt (blue) or Cdk1-AF (red). n > 100 cells were analyzed for each experimental condition. (C) Quantification of Cdk1 activation over time in cells expressing Cdc25C-wt (blue) or Cdc25C-CD (C377S) (red). The time courses of individual cells were fitted as described in Figure 4C. Rise times (τ) were calculated from the curve fits for all cells and are expressed as means ± SD. n > 20 cells in each condition. (D) Duration of mitosis in cells ectopically expressing Cdc25C-wt (blue) or Cdc25C-CD (red). n > 100 cells were analyzed for each experimental condition. (E) Quantification of Cdk1 activation over time in single cells in the absence (blue) or presence of leptomycin B (red). (F) Duration of mitosis absence (blue) or presence of leptomycin B (red). n > 100 cells were analyzed for each experimental condition. (G) Duration of mitosis as measured by the time between NEB and NER and the onset of NEB in cells expressing Cdk1-wt (blue) or Cdk1-AF (red). The trend lines are shown (blue line: m = 0.0016, R^2^ = 0.043, r = 0.21 and red line: m = 0.017, R^2^ = 0.30, r = 0.54). n > 100 cells were analyzed for each experimental condition. (H) Duration of mitosis as measured by the time between NEB and NER and the onset of NEB in cells expressing Cdc25C-wt (blue) or Cdc25C-CD (C377S) (red). The trend lines are shown (blue line: m = 0.0027, R^2^ = 0.041, r = 0.21 and red line: m = 0.019, R^2^ = 0.29, r = 0.54). n > 100 cells were analyzed for each experimental condition. (I) Duration of mitosis as measured by the time between NEB and NER and the onset of NEB in the absence (blue) or presence of leptomycin B (red). The trend lines are shown (blue line: m = 0.0039, R^2^ = 0.041, r = 0.20 and red line: m = 0.038, R^2^ = 0.24, r = 0.49). n > 100 cells were analyzed for each experimental condition.

Similar results were also obtained when we perturbed feedback regulation that leads to both activation and spatial redistribution of Cdk1 in different ways: by expressing Cdk1-AF, a form of Cdk1 which cannot be phosphorylated by Wee1 (Figures 5A, 5B, and 5G); by expressing Cdc25C-C337S, a catalytic dead Cdc25C phosphatase (Figures 5C, 5D, and 5H); and by treating cells with leptomycin B, perturbing spatial positive feedback (as described in Santos et al., 2012) (Figures 5E, 5F, and 5I). As described previously, all these perturbations of feedback regulation render Cdk1 activation more graded (Figures 5A, 5C, and 5E), duration of mitosis longer and more variable (Figures 5B, 5D, and 5F), and couple duration of mitosis to cell-cycle length (Figures 5G–5I). Similar results were also seen in other cells (Figure S5).

Taken together, these results suggest that positive feedback is important to keep mitotic events synchronized and that duration of mitosis is insulated from duration of earlier cell-cycle phases. This strongly suggests that positive feedback regulation may underlie temporal modularity in mitosis.

### SAC Does Not Contribute to Temporal Insulation of Mitosis

**Figure 6:**
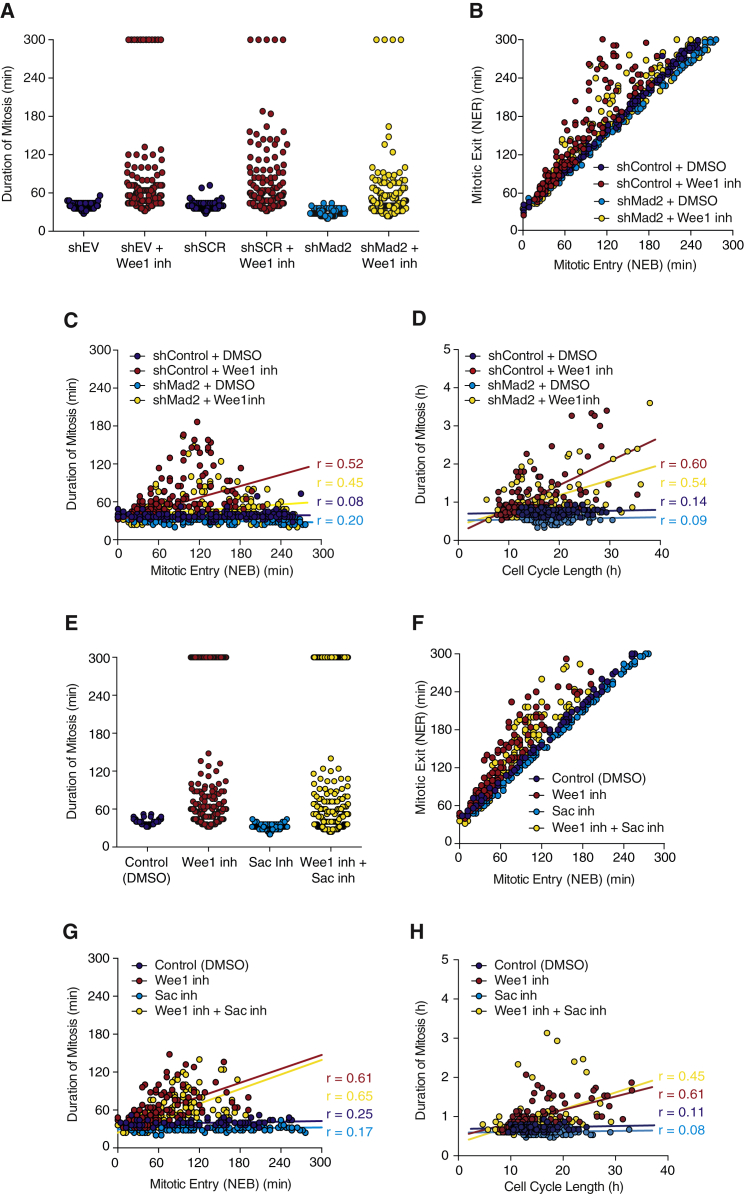
SAC Does Not Contribute to Duration of Mitosis Being Temporally Insulated from Duration of Upstream Cell-Cycle Events (A) Duration of mitosis (measured by the time between NEB and NER) in the presence or absence of Wee1 inhibitor in single cells stably expressing shEmpty vector or shScramble as controls or shMad2 for SAC inhibition. n > 115 cells were analyzed for each experimental condition. (B) Time of mitotic exit (NER) as a function of time of entry into mitosis (NEB) in control (shScramble) and SAC perturbed (shMad2) cells. The cells were either treated with DMSO or Wee1 inhibitor. n > 100 cells were analyzed for each experimental condition. (C) Duration of mitosis as a function of mitotic entry (NEB) in control (shScramble) and SAC perturbed (shMad2) cells. The cells were either treated with DMSO or Wee1 inhibitor. n > 100 cells were analyzed for each experimental condition. The trend lines are shown. (dark blue line: m = 0.0054, R^2^ = 0.0061, r = 0.078; light blue line: m = 0.014, R^2^ = 0.054, r = 0.020; red line: m = 0.30, R^2^ = 0.20, r = 0.52; and yellow line: m = 0.077, R^2^ = 0.054, r = 0.45). (D) Duration of mitosis as a function of cell-cycle length in control (shScramble) and SAC perturbed (shMad2) cells. The cells were either treated with DMSO or Wee1 inhibitor. n > 100 cells were analyzed for each experimental condition. The trend lines are shown. (dark blue line: m = 0.0027, R^2^ = 0.0019, r = 0.14; light blue line: m = 0.0021, R^2^ = 0.0076, r = 0.087; red line: m = 0.061, R^2^ = 0.36, r = 0.60; and yellow line: m = 0.040, R^2^ = 0.030, r = 0.54). (E) Duration of mitosis in single cells treated with SAC inhibitor (Sac inh) in the presence or absence of Wee1 inhibitor. DMSO was used as a control. n > 100 cells were analyzed for each experimental condition. (F) Time of mitotic exit (NER) as a function of time of entry into mitosis (NEB) in control (DMSO) and SAC inhibitor treated cells in the presence or absence of Wee1 inhibitor. n > 200 cells were analyzed for each experimental condition. (G) Duration of mitosis as a function of mitotic entry (NEB) in control (DMSO) and SAC inhibited (Sac inh) cells. The cells were either treated with DMSO or Wee1 inhibitor. n > 100 cells were analyzed for each experimental condition. The trend lines are shown. (dark blue line: m = 0.016, R^2^ = 0.062, r = 0.25; light blue line: m = 0.0098, R^2^ = 0.03, r = 0.17; red line: m = 0.37, R^2^ = 0.37, r = 0.61; and yellow line: m = 0.38, R^2^ = 0.43, r = 0.65). (H) Duration of mitosis as a function of cell-cycle length in control (DMSO) and SAC inhibited (Sac inh) cells. The cells were either treated with DMSO or Wee1 inhibitor. n > 100 cells were analyzed for each experimental condition. The trend lines are shown. (dark blue line: m = 0.0025, R^2^ = 0.012, r = 0.11; light blue line: m = 0.0014, R^2^ = 0.006, r = 0.077; red line: m = 0.034, R^2^ = 0.37, r = 0.61; and yellow line: m = 0.043, R^2^ = 0.21, r = 0.45).

In order to rule out the contribution of SAC to the temporal insulation of mitosis, we next tested the effect of compromising positive feedback in the absence of checkpoint activation. We perturbed checkpoint activation either by using cell lines stably expressing shMad2, as described above, or by using a specific SAC inhibitor, Reversine, which targets the kinase Mps1 (Santaguida et al., 2010). Mitotic duration was measured in MCF10A cells either treated with DMSO or Myt1/Wee1 inhibitor PD 166285 in the absence (shMad2) or presence (shEmpty vector or shScramble) of checkpoint control. As seen previously, in control cells, mitosis is shorter when SAC is inhibited (shMad2 28.9 ± 4.6 min, shEmpty vector 37.5 ± 4.2 min, and shScramble 37.9 ± 5.1 min), but mitotic duration remains constant. By treating cells with Myt1/Wee1 inhibitor, positive feedback is perturbed and mitosis becomes longer and more variable in both control and SAC inhibited cells (Figure 6A). In addition, SAC inhibition alone does not interfere with synchronicity between early and late mitotic events (Figure 6B). Only when positive feedback is perturbed does the timing between early and late mitotic events become unsynchronized (Figure 6B). Importantly, SAC inhibition alone does not uncouple the timing of mitotic entry from duration of mitosis. In the presence or absence of SAC, timing of mitotic entry or overall cell-cycle length and duration of mitosis are only correlated when positive feedback is compromised (Figures 6C and 6D). Similar results were seen in RPE and HeLa cells (Figure S6). We further repeated these experiments in the presence of the SAC inhibitor reversine and obtained strikingly similar results (Figures 6E–6H). Moreover, measurements performed in RPE and HeLa cells showed similar outcomes (Figure S6).

These results suggest that positive feedback alone and not SAC activation ensures mitosis is short, constant, and temporally uncoupled from earlier cell-cycle events.

### ODE Model Predicts Positive Feedback Brings about Temporal Modularity in Mitosis

**Figure 7:**
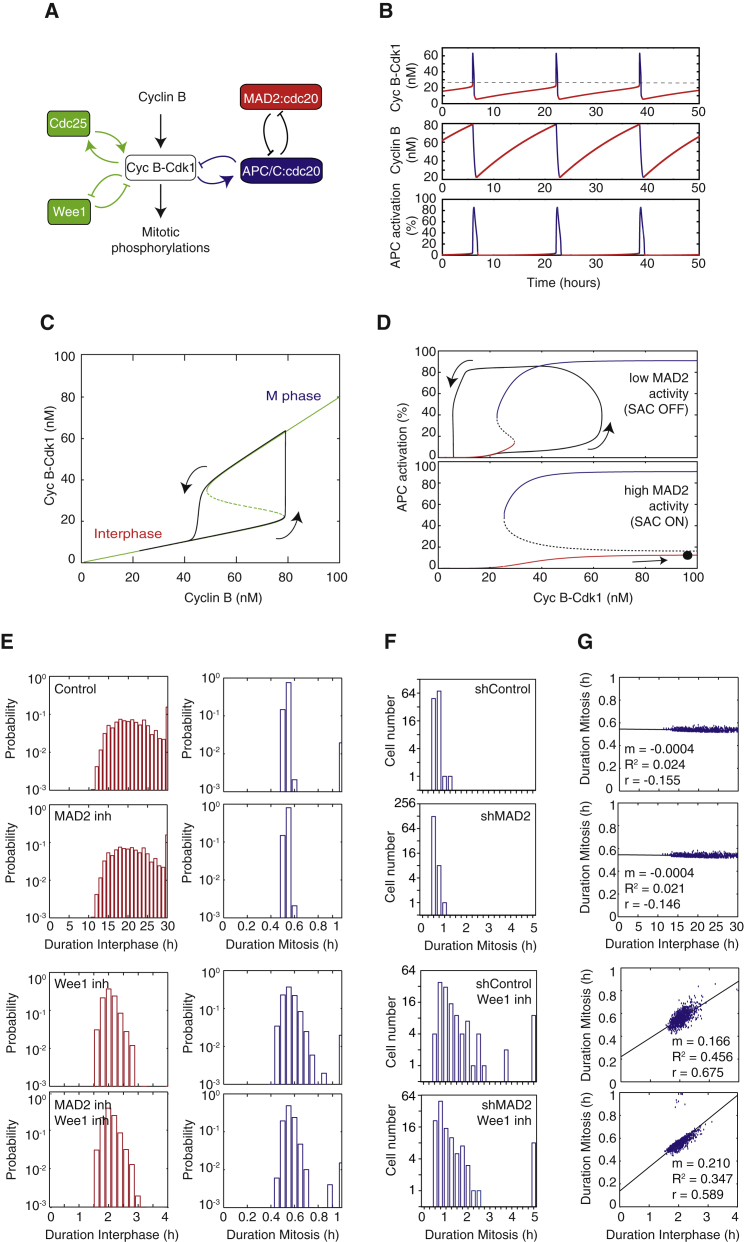
ODE Modeling Predicts that Positive Feedback Promotes Temporal Modularity in Mitosis (A) Wiring diagram showing a simplified Cdk1 regulatory network, including positive and negative feedback loops. (B) Simulation of the time evolution of the concentration of active Cdk1-cyclin B1 complexes (top), the concentration of Cyclin B (middle), and APC-cdc20 activation (bottom). (C) Steady-state responses of Cdk1-cyclin B1 activation as a function of Cyclin B1 concentration. The positive and double-negative feedback loops involving Cdc25, Wee1, and Cdk1-cyclin B1 (A, green) can give rise to bistability. (D) Steady-state responses of APC-cdc20 activation as a function of active Cdk1-cyclin B1 concentration. The double-negative feedback loop involving APC-cdc20 and MAD2-cdc20 (A, blue/red) can give rise to bistability. (E) Simulated probability distribution function (in log scale) of the duration of interphase (left panel) and mitosis (right panel) in control cells (control), with inhibition of SAC (MAD2 inh), with inhibition of the positive feedback loops involving Wee1 (Wee1 inh), or with inhibition of both SAC and Wee1 positive feedback loops (MAD2 inh + Wee1 inh). n = 100 cells were simulated for each condition. (F) Histograms (in log scale) showing experimentally measured duration of mitosis in control (shScramble), with inhibition of SAC (MAD2 inh), with inhibition of the positive feedback loops involving Wee1 (Wee1 inh), or with inhibition of both SAC and Wee1 positive feedback loops (MAD2 inh + Wee1 inh). n > 120 cells were analyzed for each experimental condition. (G) Simulated duration of mitosis as a function of duration of interphase for control cells (control), with inhibition of SAC (MAD2 inh), with inhibition of the positive feedback loops involving Wee1 (Wee1 inh), or with inhibition of both SAC and Wee1 positive feedback loops (MAD2 inh + Wee1 inh). The trend lines and associated slopes, m, R^2^ and Pearson r, are shown.

We next set out to determine whether positive feedback could plausibly be expected to insulate mitosis from previous cell-cycle events and thereby generate modularity. To this end, we formulated a simple model of ordinary differential equations (ODEs) describing the basic regulatory network that drives cells in and out of mitosis (Figure 7A, see Supplemental Informationfor details). We implemented a set of deterministic ODEs with a noisy parameter variation from cell to cell rather than a stochastic Gillespie model because the number of Cdk1-cyclin B1 complexes in a somatic cell is large (≈1.6 × 10^6^ (Sun et al., 2010). The final model used consists of three ODEs as follows:

$$\frac{d\left[Cdk1^{*}\right]\left(t\right)}{dt}=k_{s}-a_{\deg}\left[Cdk1^{*}\right]\left(t\right)-b_{\deg}\frac{\left[APC\right]{\left(t\right)}^{n_{apc}}}{EC50_{apc}^{n_{apc}}+\left[APC\right]{\left(t\right)}^{n_{apc}}}\left[Cdk1^{*}\right]\left(t\right)+\left(a_{cdc}+b_{cdc}\frac{\left[Cdk1^{*}\right]{\left(t\right)}^{n_{cdc}}}{EC50_{cdc}^{n_{cdc}}+\left[Cdk1^{*}\right]{\left(t\right)}^{n_{cdc}}}\right)\left(\left[cycB\right]\left(t\right)-\left[Cdk1^{*}\right]\left(t\right)\right)-\left(a_{wee}+b_{wee}\frac{EC50_{wee}^{n_{wee}}}{EC50_{wee}^{n_{wee}}+\left[Cdk1^{*}\right]{\left(t\right)}^{n_{wee}}}\right)\left[Cdk1^{*}\right]\left(t\right)$$

$$\frac{d\left[cycB\right]\left(t\right)}{dt}=k_{s}-a_{\deg}\left[cycB\right]\left(t\right)-b_{\deg}\frac{\left[APC\right]{\left(t\right)}^{n_{apc}}}{EC50_{apc}^{n_{apc}}+\left[APC\right]{\left(t\right)}^{n_{apc}}}\left[cycB\right]\left(t\right)$$

$$\frac{d\left[APC\right]\left(t\right)}{dt}=\left(a_{K}+b_{K}\frac{\left[CycB-Cdk1\right]{\left(t\right)}^{n_{K}}}{\left[CycB-Cdk1\right]{\left(t\right)}^{n_{K}}+EC50_{K}^{n_{K}}}\right)\left(1-\left[APC\right]\left(t\right)\right)-\left(a_{P}+b_{P}\frac{EC50_{P}^{n_{P}}}{\left[APC\right]{\left(t\right)}^{n_{P}}+EC50_{P}^{n_{P}}}\right)\left[APC\right]\left(t\right)$$

Where the following parameters were chosen: κ_s_ = 0.1 nM min^−1^, *a*_deg_ = 0.001 min^1^, *b*_deg_ = 0.02 min^−1^, a_cdc_ = 0.5 min^−1^, *b*_cdc_ = 1.5 min^−1^, EC_50cdc_ = 30 nM, n_cdc_ = 10, *a*_wee_ = 0.5 min^−1^, *b*_wee_ = 1 min^−1^, EC_50wee_ = 30 nM, n_wee_ = 10, EC_50apc_ = 0.5, n_apc_ = 10, *a*_K_ = 0 min^−1^, *b*_K_ = 0.25 min^−1^, EC_50K_ = 0.18, nK = 5, *a*_P_ = 0.025 min^−1^, *b*_P_ = 0.5 min^−1^, EC_50P_ = 0.18, and n_P_ = 5.

We first used the model to simulate the time evolution of the concentrations of active Cdk1-cyclin B1 complexes, of Cyclin B1, and of active APC-cdc20 (Figure 7B). The corresponding steady-state response of active Cdk1-cyclin B1 as a function of Cyclin B1 accumulation is also shown in Figure 7C. For intermediate values of Cyclin B concentration, two stable solutions exist (represented as a solid green line): high Cdk1 activity (M-phase) and low Cdk1 activity (interphase). One unstable solution (represented as a dashed green line) serves as a critical threshold value of Cdk1 activity, below which the system will switch to interphase and above which the system will switch to mitosis (Figure 7C). The solid black line corresponds to the trajectory in the (Cyclin B, active Cdk1-cyclin B1) plane of the cell-cycle oscillations toggling between M-phase and interphase, as shown in Figure 7B. We also show the steady-state response of APC-cdc20 activation as a function of Cdk1-cyclin B1 concentration (Figure 7D). The double-negative feedback loop involving APC-cdc20 and MAD2-cdc20 can give rise to bistability. For intermediate values of active Cdk1-cyclin B1 concentration, two stable solutions exist: high APC activity (represented as a solid blue line) and low APC activity (represented as a solid red line). The dashed line again shows the threshold value between both solutions. The region of bistability is greatly extended when MAD2 activity is increased (normal versus high MAD2 activity) (Figure 7D). Such high Mad2 activity prevents APC-Cdc20 activation when Cdk1-cyclin B1 increases, similar to when SAC is active. The solid black line corresponds to the trajectory in the (active Cdk1-cyclin B1, APC-cdc20 activation) plane of the cell-cycle oscillations shown in Figure 7B. This shows that upon Cdk1-cyclin B1 (in)activation, APC-cdc20 is fairly quickly (in)activated, except when Mad2 activity is high. Next, after implementation of noise in the model (see Supplemental Experimental Procedures), we simulated the probability distribution function of the duration of interphase and mitosis in control cells, in cells where SAC was inhibited and in the presence or absence of positive feedback (Figure 7E). We observed that the duration of interphase was unchanged both in control cells and when SAC was inhibited. On the contrary, cells where positive feedback was compromised showed shorter interphase duration, as expected by the premature entry into mitosis after Myt1/Wee1 inhibition. Importantly, duration of mitosis, while short and constant in both control and SAC inhibited cells, became longer and more variable when positive feedback was compromised (Figure 7E). Remarkably, these simulations are well in line with the measured experimental data for MCF10A, RPE, and HeLa cells (Figures 4 and S4). In addition, simulating the duration of mitosis as a function of duration of interphase (an approximation of cell-cycle length) showed no correlation in control and SAC inhibited conditions (shControl, r = 0.155 and shMad2, r = 0.146, respectively), suggesting that duration of mitosis is uncoupled from duration of interphase (Figure 7G). However, when positive feedback is perturbed, in the presence or absence of SAC, duration of mitosis becomes coupled to duration of interphase, as seen by the resulting strong correlations (shControl+Wee1 inhibitor, r = 0.675 and shMad2+ Wee1 inhibitor, r = 0.589; Figure 7G). Thus, this simple model predicts that positive feedback accounts for the experimentally observed short, constant duration of mitosis in somatic cells and for temporally insulating mitotic duration from variability in duration of upstream cell-cycle events (Figure 1). The model therefore predicts that positive feedback endows mitotic regulatory networks with the potential to bring about modularity.

## Discussion

In summary, the evidence presented here argues that positive feedback, a recurrent motif in cell-cycle regulation, is a key mechanism to ensure that duration of mitosis is kept a short, constant fraction of a typically long and variable cell cycle (Figure 1). In the human somatic cell lines we tested, the length of the cell-cycle scales well with the number of chromosomes. In other words, there is some degree of correlation between chromosome number and cell-cycle duration (i.e., cells with higher number of chromosomes have longer cell-cycle lengths). This may potentially be a direct consequence of the time needed to replicate and segregate mammalian chromosomes, arguably the main function of a cell division cycle. However, at the single cell level, individual cells have highly variable cell-cycle length. We found that this was a consequence of a high variability in the dynamics of G1-, S-, and G2-phases (Figure 1). Surprisingly, this variability was not seen for mitotic duration. There was in fact no correlation between cell-cycle length and duration of mitosis in single cells (Figure 2). This suggests that it does not seem to matter how long it takes for previous cell-cycle phases to be completed, once a cell enters mitosis it decisively completes mitosis within a short, remarkably constant time. We therefore found that duration of mitosis is temporally insulated from any variability in upstream events. We suggest that these properties are unlikely to be due to checkpoint control mechanisms since compromising the activation of the spindle assembly checkpoint failed to couple duration of mitosis and cell-cycle length (Figure 3).

Our results are consistent with earlier studies in *Xenopus* extracts (Georgi et al., 2002), a system where checkpoint control is inactive. Here, the lag time between phosphorylation of Cdc25 and phosphorylation of Cdc27 (an early and a late mitotic event, respectively) was shown to be constant regardless of how long the extract stayed in interphase (Georgi et al., 2002). In other words, it did not matter how long interphase took, duration of mitosis was kept constant. In addition, the authors observed that once Cdk1 become active, the nuclear envelope broke down and chromatin condensed within the same (constant) lag time (Georgi et al., 2002). This strongly suggests that a short and constant duration of mitosis, which is uncoupled from the timing of early events, is unlikely to be specific to mammalian somatic cells, but rather a general design principle of cell-cycle regulation.

We propose that positive feedback in the networks that regulate mitosis is the molecular mechanism that insulates duration of mitosis from the measured variability in earlier cell-cycle events. Consistent with this, compromising the switch-like activation of Cdk1-cyclin B1, a master regulator of mitosis, compromised the ability of cells to complete mitosis in a timely, constant fashion and coupled duration of mitosis to duration of interphase (or overall cell-cycle length) (Figures 4 and 5). This was indeed the case regardless of spindle assembly checkpoint activation (Figure 6). Breaking positive feedback regulation causes mitosis to become considerably longer and variable in individual cells. We observe that under these circumstances cells die during mitosis or shortly after, never reaching a second round of division (Figure S5). This suggests that a short, constant mitosis, uncoupled from variability in upstream events, is a crucial property for fidelity of cell division and cell survival. Positive feedback may help and achieve proper Cdk1 activation thresholds underlying proper progression and exit from mitosis. This observation is consistent with the elegant work from Reijo-Pera and colleagues (Wong et al., 2010) on in vitro fertilized human embryos, which showed in early embryonic divisions, a constant (15 min) duration of cytokinesis was essential to ensure embryo viability and progression through development. This raises the hypothesis that a constant duration of mitosis might be advantageous for cells and that cells have actively kept a mechanism to temporally insulate mitosis and ensure cell survival.

Together with the described Cdk1 regulatory networks and the spindle assembly checkpoint, temporal control of mitosis is also regulated by mitotic phosphatases, namely members of the PP1 and PP2A families (Bollen et al., 2009). PP2A and PP1 have been implicated in both mitotic entry and exit from mitosis by reversing Cdk1 substrate phosphorylation (reviewed in Bollen et al., 2009). Due to the fact that PP1 and PP2A have been implicated in feedback regulation with Cdk1 (Burgess et al., 2010, Castilho et al., 2009, Dohadwala et al., 1994, Kwon et al., 1997), we anticipate that compromising specifically PP1 and PP2A activation will likely impact on the observed modularity of mitosis.

Computational modeling was used to test whether positive feedback could account for the observed temporal insulation of mitosis. Our model expanded previous ODE models describing the activation and deactivation cycles of Cdk1-cyclin B1 (Pomerening et al., 2003, Yang and Ferrell, 2013) by including feedback regulation between APC-cdc20 and Mad2, an important component of the spindle assembly checkpoint. Notably, the model predicted that positive feedback in the Cdk1-cyclin B1 regulatory network alone could give rise to short mitoses whose duration was uncoupled from the duration of interphase (Figure 7). As seen experimentally, compromising positive feedback compromised temporal insulation of mitosis.

One of the most important steps forward in our understanding of the systems biology of cellular decision making has been the appreciation that there are simple, ubiquitous motifs that define how genes and proteins regulate each others’ activities (Alon, 2007). Positive feedback loops are examples of such recurrent cellular strategies and have been shown to bring about amplification, maintenance, and rapid switching of activities in time and space (Ferrell, 2002, Santos et al., 2012, Chang and Ferrell, 2013, among others) and be the basis for unidirectional, coherent, and all-or-none cellular events (Ferrell and Machleder, 1998, Xiong and Ferrell, 2003, Novak et al., 2007, Holt et al., 2008, Skotheim et al., 2008, López-Avilés et al., 2009, He et al., 2011).

The work presented here shows that positive feedback regulation can generate temporal insulation and bring about modularity. In mammalian cells, coupling timing of G1-, S-, and G2-phases may endow cells with the potential to couple growth, DNA replication, and repair, all cell-cycle events that are likely to influence (and depend) on one another. A delay in cell growth during G1 might delay commitment to DNA replication. Delay in DNA replication might delay completion of DNA repair. However, when entering mitosis, cells will undergo dramatic morphological changes, stopping most metabolic and transcriptional activity in preparation for an even segregation of chromosomes. Uncoupling mitosis from earlier events might allow individual cells to avoid noise and variability of the early phases and (truly) irreversibly commit to segregating chromosomes into daughter cells in a short, fast, and all-or-none manner. Given how modularity is emerging as an important and widespread feature of biological systems (Atay and Skotheim, 2014, Hartwell et al., 1999, Nachman et al., 2007), we anticipate that positive feedback may prove to be a recurrent cellular strategy to temporally uncouple events beyond the cell-cycle field.

## Experimental Procedures

### Cell Lines

All the experiments in this study were performed in human MCF10A, RPE, and HeLa cell lines. Details on growth and maintenance of all the cell lines used can be found in Supplemental Experimental Procedures.

### Biosensors, shRNAs, and Establishment of Stable Lines

cDNAs for Histone H2B fused to Cerulean, Cdt1 (amino acid [aa], 30–120) (Sakaue-Sawano et al., 2008) fused to YFP, PCNA fused with RFP (Sporbert et al., 2005), Cyclin B1 fused with YFP (Santos et al., 2012), Cdk1-AF, Cdk1-wt (Santos et al., 2012), Cdc25C-wt, and Cdc25C-Catalytic dead (C377S) (Santos et al., 2012), NLS (×3) fused to mCherry (Santos et al., 2012) were all cloned into the lentiviral vector CSII-EF-1-MCS-2 by restriction digestion and ligation reactions. The CSII-EF-1-MCS-2 plasmid is a modified CSII-EF-1-MCS backbone vector where a linker TCGAAGCTAGCCCTGCAGGTTAATTAAC has been added to the MCS to increase the number of unique restriction sites. Stable MCF10A, RPE, and HeLa cells lines were made with the following combination of cell-cycle biosensors: Cdt1-YFP, PCNA-mCherry, and H2B-Cerulean or Cyclin B1-YFP and NLS3-mCherry and H2B-CFP. Lentivirus production was carried out in 293T cells transfected with DNA of interest and lentivirus assembly vectors (PAX2 and VSV-G) using with Polyethylenimine (PEI). Cells were infected for 12 hr using polybrene (8 μg). 72 hr post-infection, transduced cells were sorted on a Becton Dickinson FACSAria III influx to obtain pure populations expressing the desired fluorescent reporters. For creation of shMad2 stable lines, a set of two shRNA (GIPZ lentiviral shRNA Pool, Dharmacon, Thermo Scientific) specific to Mad2 in lentiviral constructs were used (clone V3LHS_327851: TGCTGTTGACAGTGAGCGCCTGGTTGTAGTTATCTCAAATTAGTGAAGCCACAGATGTAATTTGAGATAACTACAACCAGTTGCCTACTGCCTCGGA and clone V3LHS_403761:TGCTGTTGACAGTGAGCGCATGGATATTTGTACTGTTTAATAGTGAAGCCACAGATGTATTAAACAGTACAAATATCCATTTGCCTACTGCCTCGGA). Stable lines expressing shEmpty (pGIPZ, Dharmacon, Thermo Scientific) vector and shScramble (GIPZ non-silencing shRNA control. Sequence: TGCTGTTGACAGTGAGCGATCTCGCTTGGGCGAGAGTAAGTAGTGAAGCCACAGATGTACTTACTCTCGCCCAAGCGAGAGTGCCTACTGCCTCGGA) were used as controls for experiments with shMad2. MCF10A, RPE, and HeLa cells were infected with the pool of two shRNAs. Transduced cells were selected with 2 μg/mL of puromycin.

### Inhibitors

The inhibitors used in this study were: Wee1/Myt1 inhibitor, PD 166285, (at 0.5 μM, 1 μM, and 2 μM), SAC inhibitor, Reversine, (at 1 μM), and Leptomycin B (at 100 ng/mL).

### Microscopy and Data Analysis

Live cell imaging was performed on either ScanR, a fully motorized and automated inverted epifluorescence microscope system IX83 (Olympus) combined with cellVivo (Olympus) or IncuCyte Zoom (Essen BioScience). Both equipped with temperature, humidity, and CO_2_ levels control to keep the sample integrity and perfect focus. Details of objectives and lenses used and details on imaging procedures can be found in Supplemental Information.

Image analysis was done with scripts written in Matlab (Mathworks) and ImageJ (NIH). Mann-Whitney and Kolmogrov-Smirnov tests were used to estimate p values. Trend lines, R^2^, person correlation coefficient, and mean absolute deviation were calculated using Prism6.

### Mathematical Modeling

In brief, the model used consists of three ODEs to simulate the time evolution of the total amount of active Cdk1 ([Cdk1^∗^](t)), the synthesis and destruction of the mitotic cyclins, Cyclin B ([cycB](t)), and active APC-cdc20 ([APC](t)). The detailed information on the model construction (equations and parameters used), noise implementation, and the setup of the numerical simulations can be found in the Supplemental Information.

## Author Contributions

A.R.A. performed all the experiments. L.G. performed all the simulations and wrote the associated methods. A.R.A., R.S.M.S., and S.D.M.S. analyzed the data. A.R.A. and S.D.M.S. designed the experiments. S.D.M.S. conceived the study and wrote the paper.

## Supplemental Information

Document S1. Supplemental Experimental Procedures and Figures S1–S7

Document S2. Article plus Supplemental Information
