# Supplementary material for: Positive Feedback Keeps Duration of Mitosis Temporally Insulated from Upstream Cell-Cycle Events
Source: Mol Cell. 2016 Oct 20;64(2):362–75. doi: 10.1016/j.molcel.2016.09.018 (PMC5077699; doi:10.1016/j.molcel.2016.09.018)
Supplement: Document S1. Supplemental Experimental Procedures and Figures S1–S7 [file mmc1.pdf]

Molecular Cell, Volume *64*

Supplemental Information

Positive Feedback Keeps Duration of Mitosis Temporally Insulated from Upstream Cell-Cycle Events

Ana Rita Araujo, Lendert Gelens, Rahuman S.M. Sheriff, and Silvia D.M. Santos

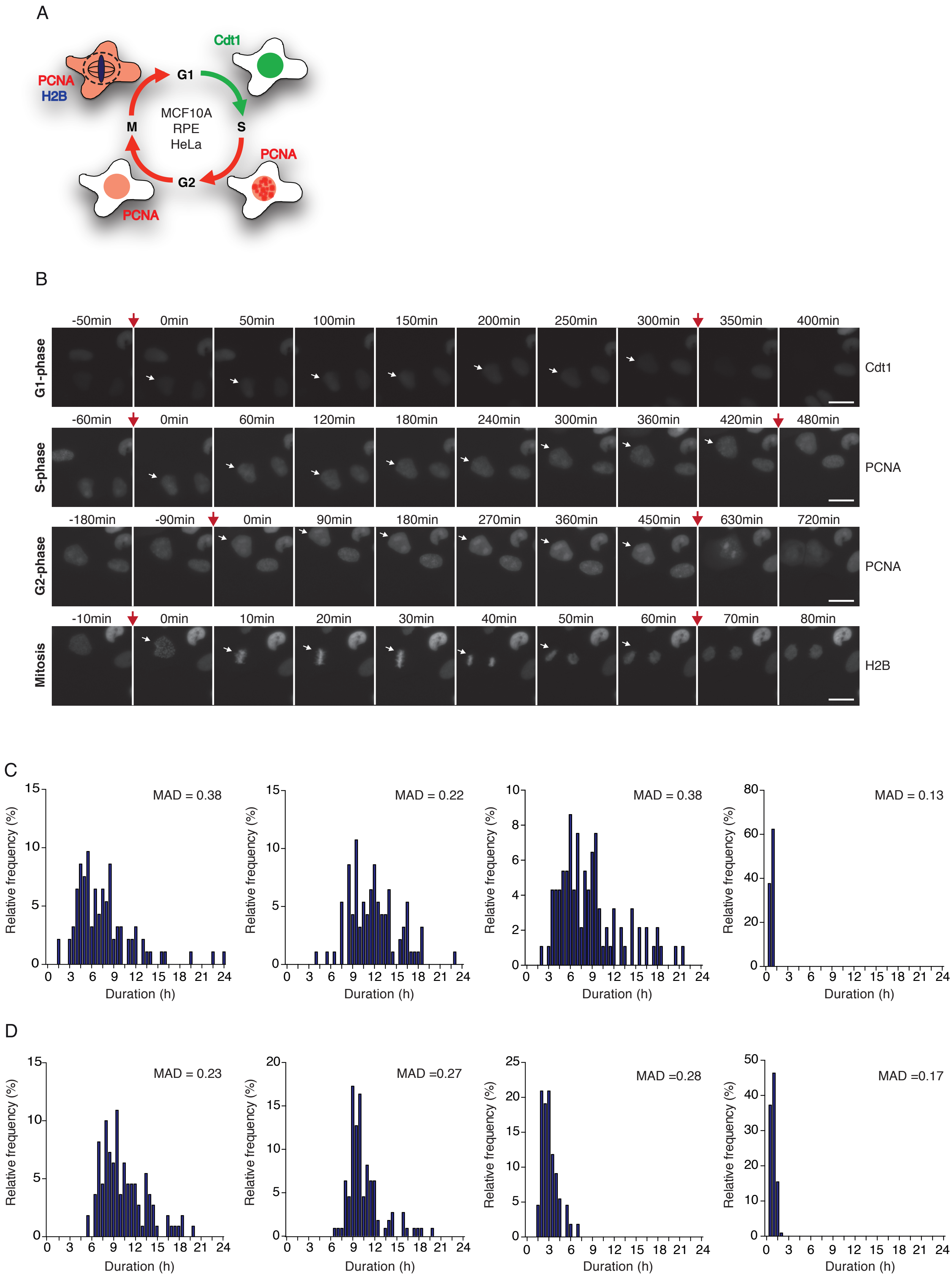

Supplemental Figure S1. Araujo, Gelens, Sheriff and Santos

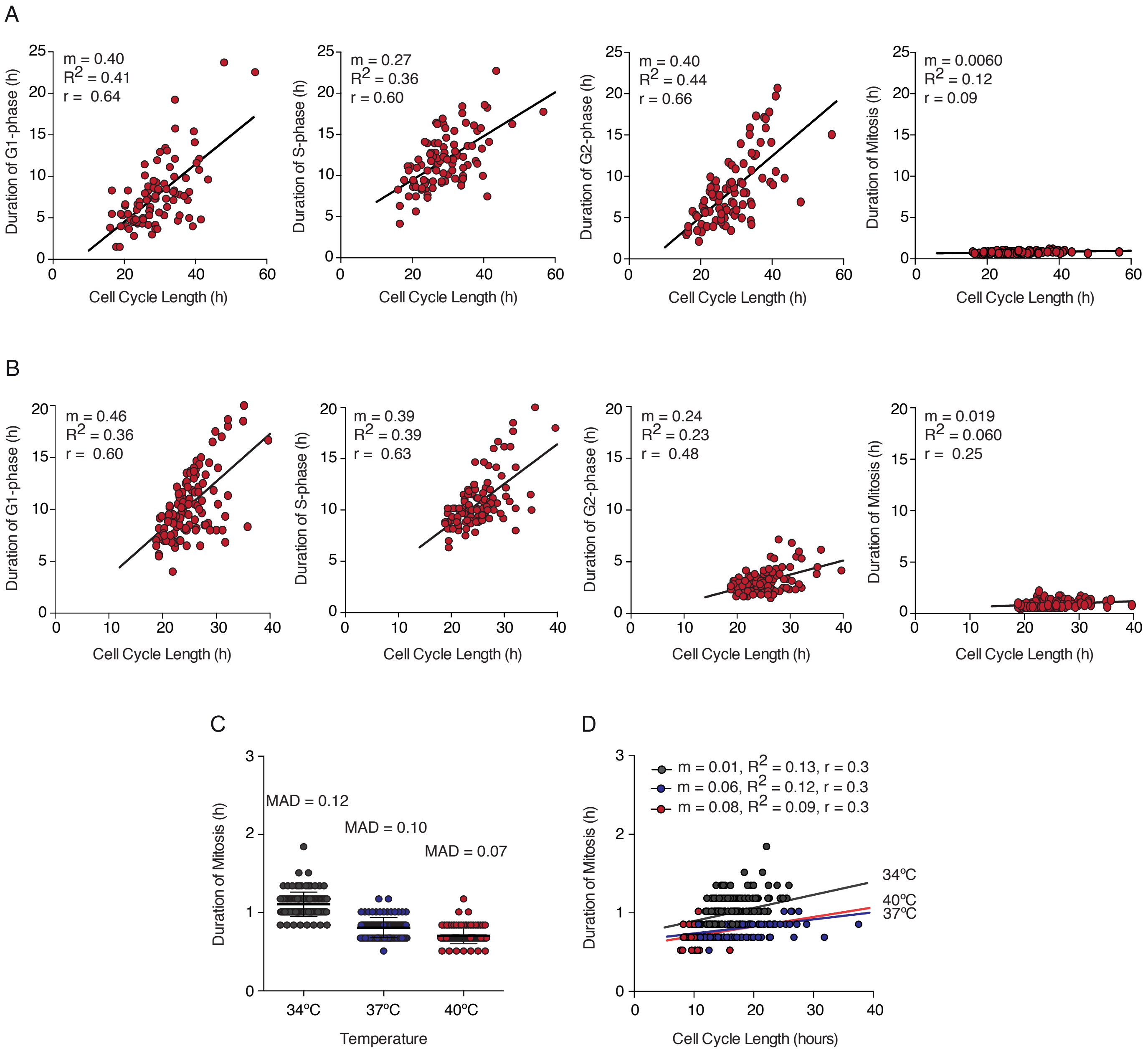

Supplemental Figure S2. Araujo, Gelens, Sheriff and Santos

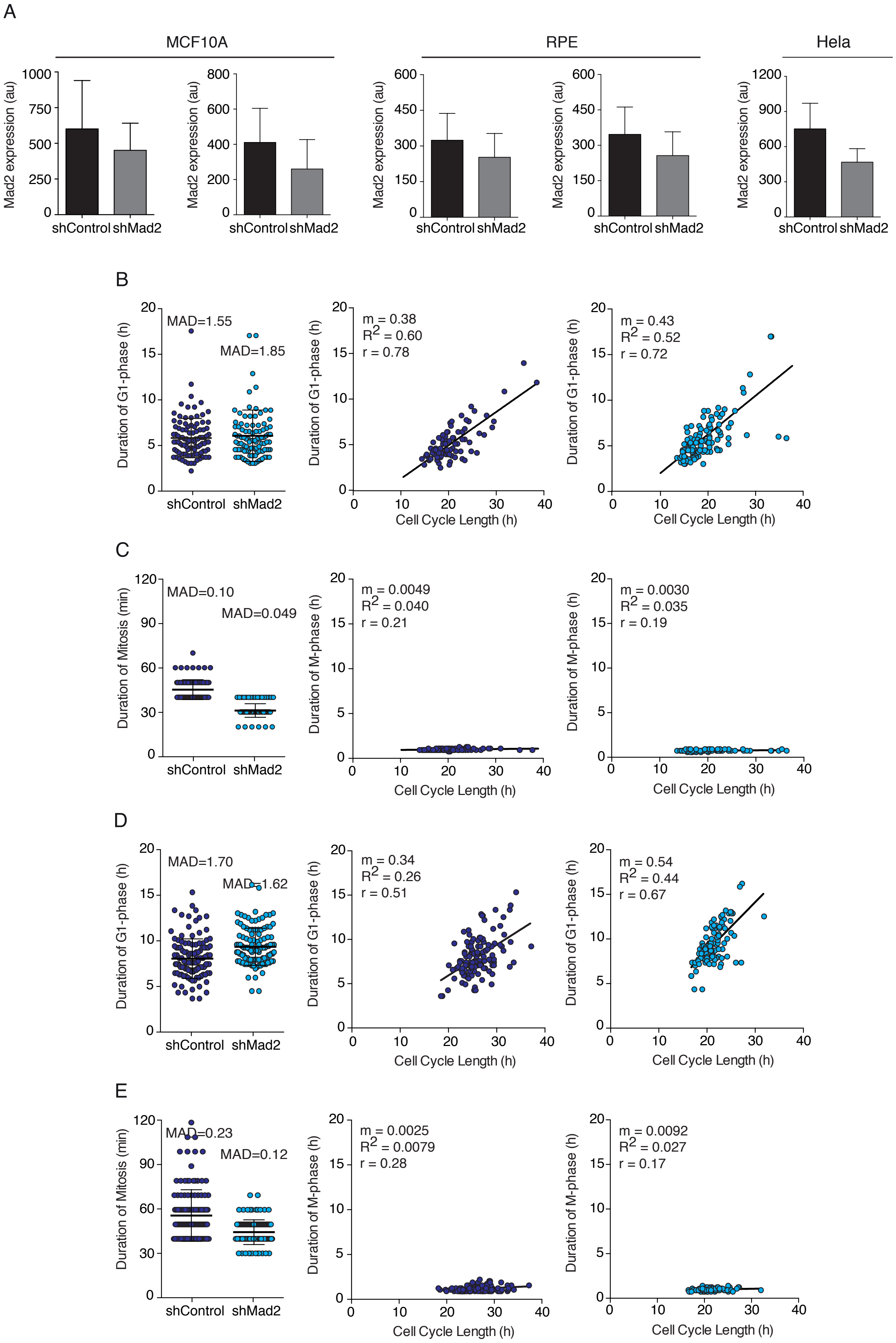

Supplemental Figure S3. Araujo, Gelens, Sheriff and Santos

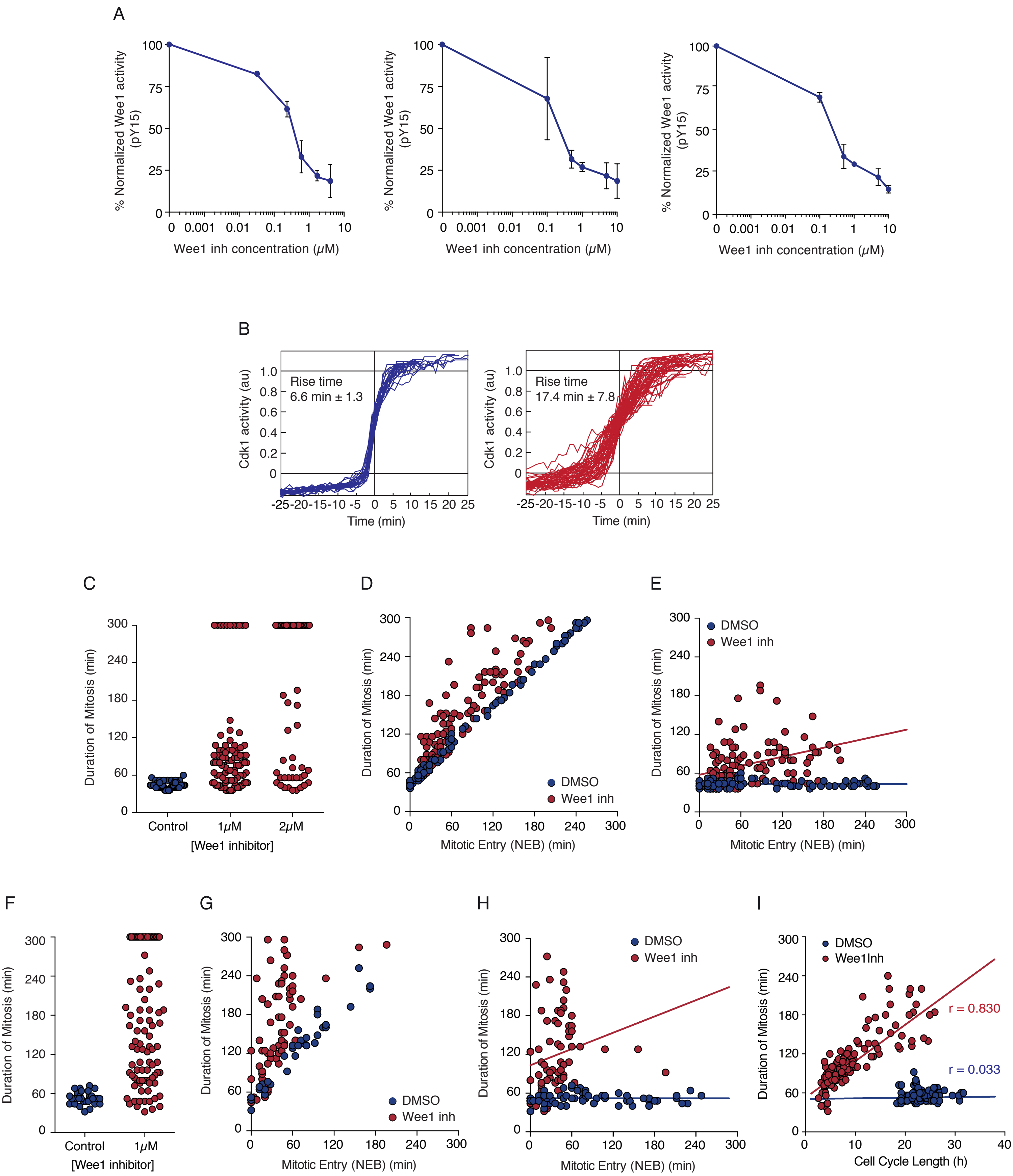

Supplemental Figure S4. Araujo, Gelens, Sheriff and Santos

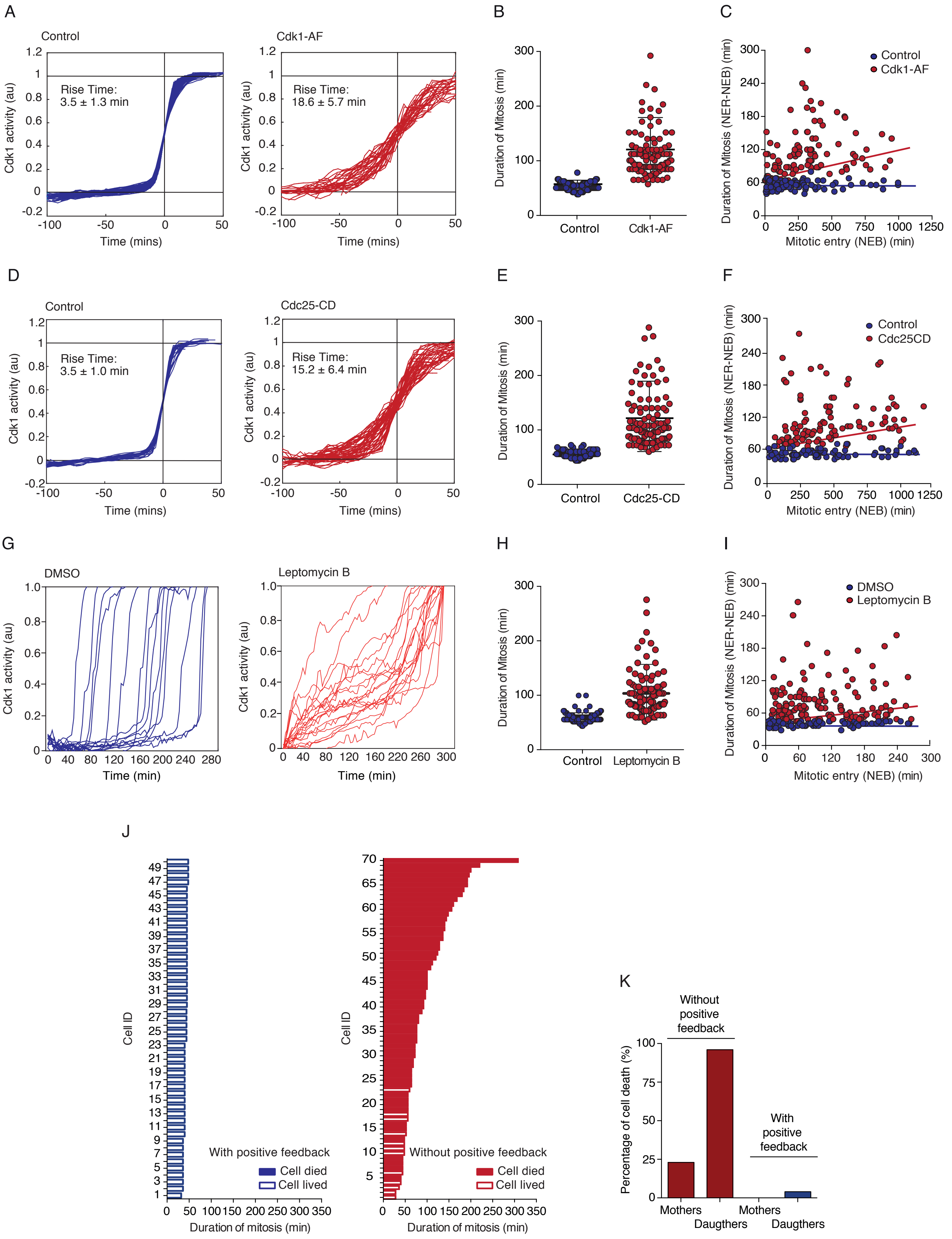

Supplemental Figure S5. Araujo, Gelens, Sheriff and Santos

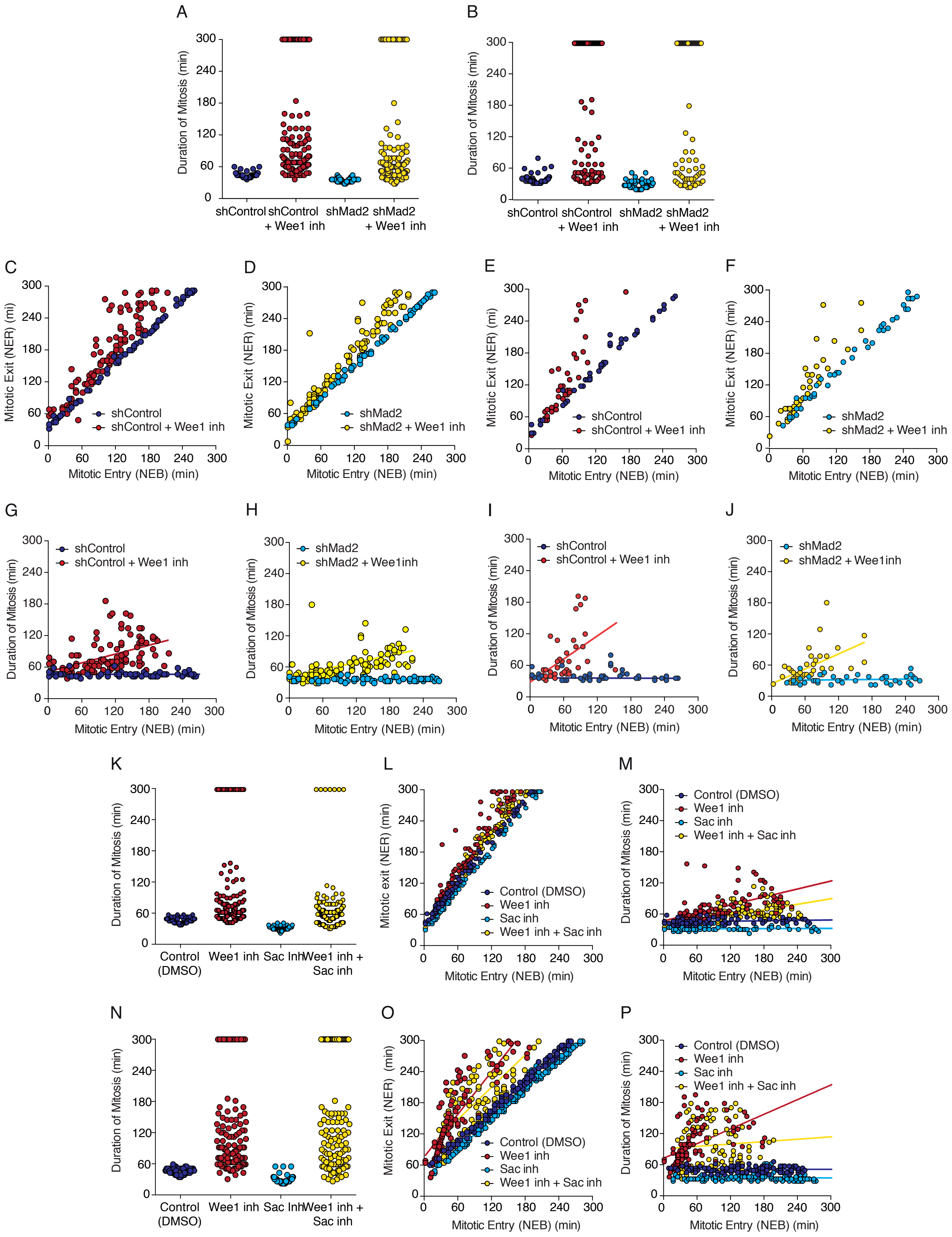

Supplemental Figure S6. Araujo, Gelens, Sheriff and Santos

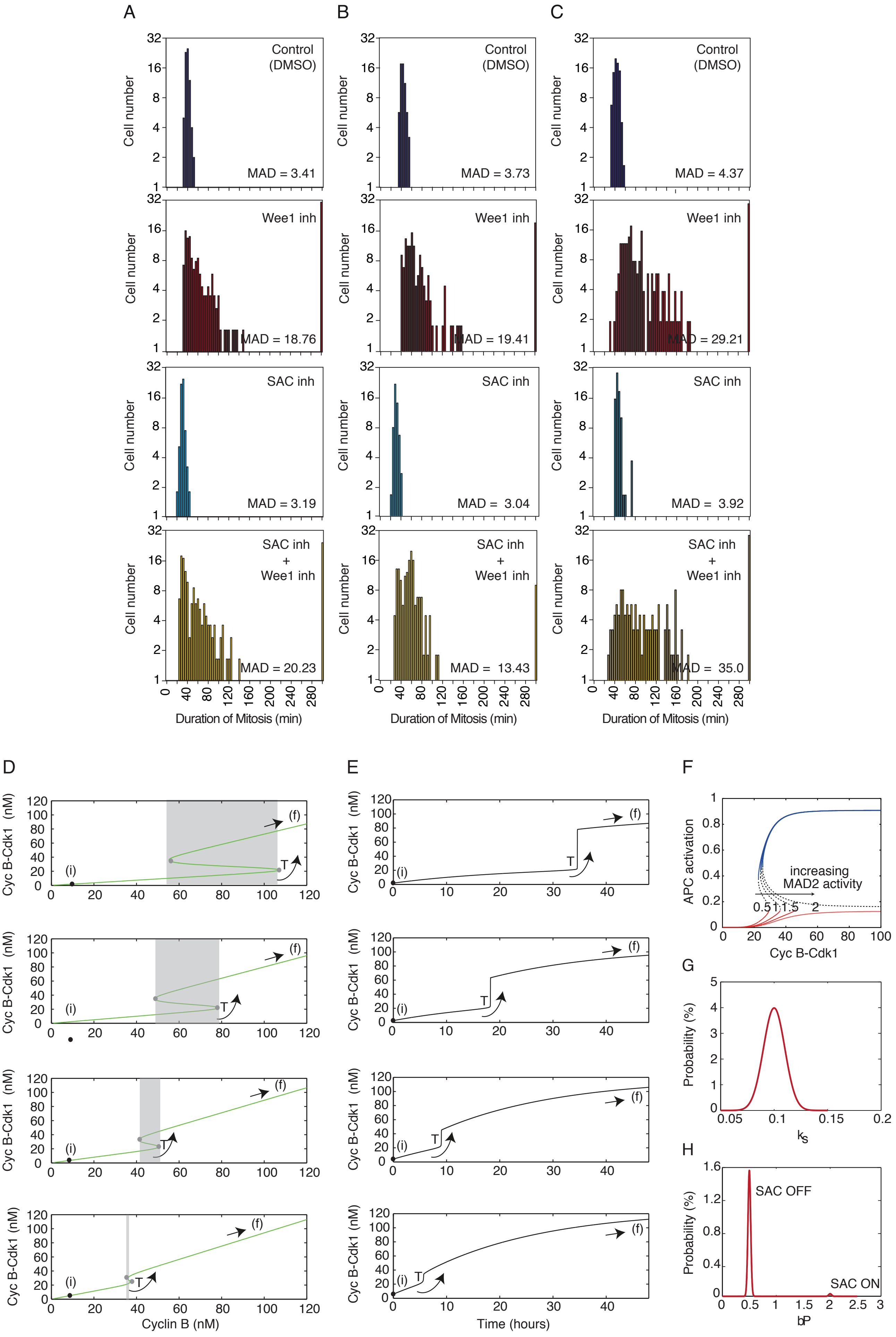

Supplemental Figure S7. Araujo, Gelens, Sheriff and Santos

Supplementary Information

Positive feedback keeps duration of mitosis temporally insulated from upstream cell cycle events

Araujo, AR, Gelens, L, Sheriff, R and Santos, SDM

Supplemental figure legends

Supplemental Figure S1 (related to Figure 1). Measuring cell cycle dynamics in single cells shows that in contrast to other cell cycle phases duration of mitosis is short and fairly constant. (A) Schematic of cell lines (MCF10A, RPE and HeLa) and biosensors (Cdt1-YFP, PCNA-mCherry and H2B-CFP) used to measure cell cycle dynamics in single cells. (B) Representative images showing how G1, S, G2 and M-phase dynamics were measured in single cells with cell cycle biosensors. Red and white arrows show duration of each cell cycle phase. For G1-phase dynamics appearance and disappearance of Cdt1 was used to measure duration. Duration of PCNA speckles was used as a proxy for S-phase length. Time between PCNA speckles disappearance and nuclear envelope breakdown was used to measure length of G2-phase. Duration of mitosis was measured by the time between nuclear envelope breakdown and nuclear envelope reformation, seen both using H2B-CFP and PCNA-mCherry re-distribution. Scale bar 10μm.
(C) Histograms showing duration of G1, S, G2 and M-phases in RPE cells. Normalized mean absolute deviations (MAD) are shown. n> 90 cells for each experimental condition. (D) Histograms showing duration of G1, S, G2 and M-phases in HeLa cells. Normalized mean absolute deviations (MAD) are shown. n> 100 cells for each experimental condition.

Supplemental Figure S2 (related to Figure 2). Duration of mitosis does not correlate with variability in cell cycle length. (A) Duration of G1, S, G2 and M-cell cycle phases in single RPE cells as a function of cell cycle length measured by live cell imaging. Trend lines (with respective slope (m) and R-squared (R^2)) and *Pearson* correlation coefficients (r) are shown. n> 90 cells for each experimental condition. (B) Duration of G1, S, G2 and M-cell cycle phases in single HeLa cells as a function of cell cycle length measured by live cell imaging. Trend lines (with

respective slope (m) and R-squared (R^2)) and *Pearson* correlation coefficients (r) are shown. n> 100 cells for each experimental condition. (C) Duration of mitosis is constant in temperature-driven changes of cell cycle length. Duration of mitosis in RPE cells as measured by live cell imaging performed at 34°C, 37°C and 40°C. Mean ± standard deviation (65.8±9.36min, 47.9±7.7min, 41.8±6.17min for 34°C, 37°C and 40°C, respectively) and mean absolute deviations (MAD) are shown. n>90 cells were analysed for each experimental condition. (D) Duration of mitosis in RPE cells as a function of cell cycle length measured at 34°C, 37°C and 40°C. Trend lines with corresponding slope (m) and R-squared (R^2) are shown. *Pearson* correlation coefficients (r) were calculated. n>90 cells were analysed for each experimental condition.

Supplemental Figure S3 (related to Figure 3). Perturbing the spindle assembly checkpoint (SAC) does not make duration mitosis variable or dependent on cell cycle length. (A) Mean expression of Mad2 in MCF10A, RPE and HeLa cells stably expressing shMad2. Two combinations of each cell line were made and used in this study: shMad2 and shScramble (shControl) together with either NLS-mCherry (left panels) or PCNA-mCherry (right panels). Standard deviation is shown for each sample. n>5000 cells were analysed for each experimental condition. (B) Left panel: Duration of G1-phase measured in RPE cells in the presence (shControl) and absence (shMad2) of SAC. shScramble was used as control (shControl). Mean ± standard deviation and mean absolute deviations (MAD) are shown (shControl 5.7±2.2h; shMad2 6.0±2.8h). Middle panel: Duration of G1-phase in control cells plotted as a function of cell cycle length. Trend lines with corresponding slope (m) and R-squared (R^2) are shown. *Pearson* correlation coefficients (r) were calculated. Right panel: Duration of G1-phase in SAC perturbed (shMad2) cells plotted as a function of cell cycle length. Trend lines with corresponding slope (m) and R-squared (R^2) are shown. *Pearson* correlation coefficients (r) were calculated. n>80 cells were analysed for each experimental condition. (C) Left panel: Duration of mitosis measured in RPE cells in the presence (shControl) and absence of SAC (shMad2). shEmpty vector was used as control (shControl). Mean ± standard deviation and mean absolute deviations (MAD) are shown (shControl 43.6±6min; shMad2 31.1±4.6min).

Middle panel: Duration of mitosis in control cells plotted as a function of cell cycle length. Trend lines with corresponding slope (m) and R-squared (R^2) are shown. *Pearson* correlation coefficients (r) were calculated. Right panel: Duration of G1-phase in SAC perturbed (shMad2) cells plotted as a function of cell cycle length. Trend lines with corresponding slope (m) and R-squared (R^2) are shown. *Pearson* correlation coefficients (r) were calculated. n>80 cells were analysed for each experimental condition. (D) Left panel: Duration of G1-phase measured in HeLa cells in the presence (shControl) and absence (shMad2) of SAC. shScramble was used as control (shControl). Mean ± standard deviation (shControl 8.0±2.2h; shMad2 9.0±2.1h) and mean absolute deviations (MAD) are shown. Middle panel: Duration of G1-phase in control cells plotted as a function of cell cycle length. Trend lines with corresponding slope (m) and R-squared (R^2) are shown. *Pearson* correlation coefficients (r) were calculated. Right panel: Duration of G1-phase in SAC perturbed (shMad2) cells plotted as a function of cell cycle length. Trend lines with corresponding slope (m) and R-squared (R^2) are shown. *Pearson* correlation coefficients (r) were calculated. n>110 cells were analysed for each experimental condition. (E) Left panel: Duration of mitosis measured in HeLa cells in the presence (shControl) and absence of SAC (shMad2). shScramble was used as control (shControl). Mean ± standard deviation and mean absolute deviations (MAD) are shown. Middle panel: Duration of mitosis in control cells plotted as a function of cell cycle length. Trend lines with corresponding slope (m) and R-squared (R^2) are shown. *Pearson* correlation coefficients (r) were calculated. Right panel: Duration of mitosis in SAC perturbed (shMad2) cells plotted as a function of cell cycle length. Trend lines with corresponding slope (m) and R-squared (R^2) are shown. *Pearson* correlation coefficients (r) were calculated. n>110 cells were analysed for each experimental condition.

Supplemental Figure S4 (related to Figure 4). Positive feedback keeps mitosis temporally insulated from upstream cell cycle events. (A) Dose response of Wee1 inhibitor in MCF10A, RPE and Hela cells. Quantification of Wee1 activity as measured by phosphorylation of Y15 on Cdk1 to increasing concentrations of Wee1 inhibitor, PD166285 by western blot for MCF10A (left), RPE (middle) and

Hela (right) cells. Total Cdk1 was used as a loading control. DMSO treated cells were used as control (0μM) and used to normalize to 100% activity. (B) Quantification of Cdk1 activation over time in the absence (blue) or presence (red) of 1μM Wee1 inhibitor, PD166285, in RPE cells. Time courses of individual cell were fitted to the logistic equation $y = a+b/1+e^{-(t-t0/\tau)}$ and were scaled to their fitted maximum and minimum values (b and a, respectively) and half-maximal times (t0). Rise times (τ) were calculated from the curve fits for all cells and are expressed as means ± standard deviation (SD). n>20 or more cells in each condition. (C) Duration of mitosis in RPE cells in the presence (red) and absence (blue) of Wee1 inhibitor at different concentrations. n> 100 cells were analysed for each experimental condition. (D) Time of NER as a function of NEB in RPE cells either treated with DMSO (blue) or with Wee1 inhibitor (red). n> 100 cells were analysed for each experimental condition. (E) Duration of mitosis (as measured by the time between NEB and NER) as a function of NEB in RPE cells in the presence (red) or absence (blue) of Wee1 inhibitor. Trend lines are shown (blue line: m = 0.0010, R^2 = 0.0031, r = 0.017; red line: m = 0.23, R^2 = 0.12, r = 0.35). (F) Duration of mitosis in HeLa cells in the presence (red) and absence (blue) of Wee1 inhibitor (1μm). n> 100 cells were analysed for each experimental condition. (G) Time of NER as a function of NEB in Hela cells either treated with DMSO (blue) or with Wee1 inhibitor (red). n> 100 cells were analysed for each experimental condition. (H) Duration of mitosis (as measured by the time between NEB) and NER and the onset of NEB in Hela cells in the presence (red) or absence (blue) of Wee1 inhibitor. DMSO was used as control. n> 100 cells were analysed for each experimental condition. Trend lines are shown (blue line: m = 0.00001, R^2 = 0.00001, r = 0.00026; red line: m = 0.42, R^2 = 0.048, r = 0.22). (I) Duration of mitosis and cell cycle length in Hela cells in the presence (red) or absence (blue) of Wee1 inhibitor. DMSO was used as control. n> 100 cells were analysed for each experimental condition. Trend lines are shown (blue line: m = 0.0016, R^2 = 0.0011, r = 0.033; red line: m = 0.098, R^2 = 0.68, r = 0.830).

Supplemental Figure S5 (related to Figure 5). Breaking Cdk1 activation and spatial positive feedbacks couples duration of mitosis to upstream cell cycle

events in HeLa cells. (A) Quantification of Cdk1 activation over time in HeLa cells expressing Cdk1-wt (blue) or Cdk1-AF (red). Time courses of individual cells were fitted as described in figure 4C. Rise times (τ) were calculated from the curve fits for all cells and are expressed as means ± standard deviation (SD). n>20 cells in each condition. (B) Duration of mitosis in cells ectopically expressing Cdk1-wt (blue) or Cdk1-AF (red). n> 100 cells were analysed for each experimental condition. (C) Duration of mitosis (as measured by the time between NEB and NER) and the onset of NEB in cells expressing Cdk1-wt (blue) or Cdk1-AF (red). Trend lines are shown. n> 100 cells were analysed for each experimental condition. (D) Quantification of Cdk1 activation over time in HeLa cells expressing Cdc25C-wt (blue) or Cdc25C-CD (C377S) (red). Time courses of individual cells were fitted as described in figure 4C. Rise times (τ) were calculated from the curve fits for all cells and are expressed as means ± standard deviation (SD). n>20 cells in each condition. (E) Duration of mitosis in cells ectopically expressing Cdc25C-wt (blue) or Cdc25C-CD (red). n> 100 cells were analysed for each experimental condition. (F) Duration of mitosis (as measured by the time between NEB and NER) and the onset of NEB in cells expressing Cdc25C-wt (blue) or Cdc25C-CD (C377S) (red). Trend lines are shown. n> 100 cells were analysed for each experimental condition. (G) Quantification of Cdk1 activation over time in HeLa cells in the absence (blue) or presence of leptomycin B (red). (H) Duration of mitosis absence (blue) or presence of leptomycin B (red). n> 100 cells were analysed for each experimental condition (I) Duration of mitosis as measured by the time between NEB and NER and the onset of NEB in the absence (blue) or presence of leptomycin B (red). Trend lines are shown. n> 100 cells were analysed for each experimental condition. (J) Perturbing positive feedback results in a long, variable duration of mitosis and leads to cell death. Quantification of mitotic duration for individual cells and cell survival after mitosis in the presence (left) or absence (right) of positive feedback. (K) Percentage of cells that died during first mitosis (mothers) or right after the first mitosis (daughters) when treated with DMSO (with positive feedback) or with 1μM Wee1 inhibitor (without positive feedback).

Supplemental Figure S6 (related to Figure 6). SAC does not contribute to duration of mitosis being temporally insulated from duration of upstream cell cycle events in RPE and HeLa cells. (A) Duration of mitosis in the presence of absence of Wee1 inhibitor in RPE cells stably expressing shScramble as control or shMad2 for SAC inhibition. n=100 cells were analysed for each experimental condition. (B) Duration of mitosis in the presence of absence of Wee1 inhibitor in HeLa cells stably expressing shScramble as control or shMad2 for SAC inhibition. n=100 cells were analysed for each experimental condition. (C) Time of mitotic exit (NER) as a function of time of entry into mitosis (NEB) in control (shScramble) cells. RPE cells were either treated with DMSO (dark blue) or Wee1 inhibitor (red). n= 100 cells were analysed for each experimental condition. (D) Time of mitotic exit (NER) as a function of time of entry into mitosis (NEB) in SAC inhibited (shMad2) cells. RPE cells were either treated with DMSO (light blue) or Wee1 inhibitor (yellow). n= 100 cells were analysed for each experimental condition. (E) Time of mitotic exit (NER) as a function of time of entry into mitosis (NEB) in control (shScramble) cells. Hela cells were either treated with DMSO (dark blue) or Wee1 inhibitor (red). n= 100 cells were analysed for each experimental condition. (F) Time of mitotic exit (NER) as a function of time of entry into mitosis (NEB) in SAC inhibited (shMad2) cells. Hela cells were either treated with DMSO (light blue) or Wee1 inhibitor (yellow). n= 100 cells were analysed for each experimental condition. (G) Duration of mitosis as a function of mitotic entry (NEB) in control (shScramble) cells. RPE cells were either treated with DMSO (dark blue) or Wee1 inhibitor (red). n= 100 cells were analysed for each experimental condition. Trend lines are shown. (dark blue line: m = 0.0021, R^2 = 0.0015; *Pearson* correlation coefficient, r = 0.038; red line: m = 0.28, R^2 = 0.25; *Pearson* correlation coefficient, r = 0.50). (H) Duration of mitosis as a function of mitotic entry (NEB) in SAC inhibited (shMad2) cells. RPE cells were either treated with DMSO (light blue) or Wee1 inhibitor (yellow). n> 100 cells were analysed for each experimental condition. Trend lines are shown. (light blue line: m = 0.0041, R^2 = 0.0094; *Pearson* correlation coefficient, r = 0.096; yellow line: m = 0.22, R^2 = 0.34; *Pearson* correlation coefficient, r = 058). (I) Duration of mitosis as a function of mitotic entry (NEB) in control (shScramble) cells. HeLa cells were either treated with DMSO (dark blue) or

Wee1 inhibitor (red). n= 100 cells were analysed for each experimental condition. Trend lines are shown. (dark blue line: m = 0.000072, R^2 = 0.0000028; *Pearson* correlation coefficient, r = 0.0053; red line: m = 0.76, R^2 = 0.29; *Pearson* correlation coefficient, r = 0.53). (J) Duration of mitosis as a function of mitotic entry (NEB) in SAC inhibited (shMad2) cells. HeLa cells were either treated with DMSO (light blue) or Wee1 inhibitor (yellow). n> 100 cells were analysed for each experimental condition. Trend lines are shown. (light blue line: m = 0.010, R^2 = 0.010; *Pearson* correlation coefficient, r = 0.10; yellow line: m = 0.48, R^2 = 0.32; *Pearson* correlation coefficient, r = 056). (K) Duration of mitosis in RPE cells treated with SAC inhibitor (Sac inh) in the presence or absence of Wee1 inhibitor. DMSO was used as a control n> 100 cells were analysed for each experimental condition. (L) Time of mitotic exit (NER) as a function of time of entry into mitosis (NEB) in control (DMSO) and SAC inhibitor treated cells in the presence or absence of Wee1 inhibitor. n> 200 cells were analysed for each experimental condition. (M) Duration of mitosis as a function of mitotic entry (NEB) in control (DMSO) and SAC inhibitor treated cells in the presence or absence of Wee1 inhibitor. n> 200 cells were analysed for each experimental condition. Trend lines are shown. (dark blue line: m = 0.012, R^2 = 0.038, *Pearson* correlation coefficient, r = 0.20; red line: m = 0.26, R^2 = 0.33, *Pearson* correlation coefficient, r = 0.58; light blue line: m = 0.0030, R^2 = 0.0037, *Pearson* correlation coefficient, r = 0.061; yellow line: m = 0.18, R^2 = 0.45, *Pearson* correlation coefficient, r = 0.68). line: m = 0.18, R2 = 0.45, Pearson correlation coefficient, r = 0.68). (N) Duration of mitosis in HeLa cells treated with SAC inhibitor (Sac inh) in the presence or absence of Wee1 inhibitor. DMSO was used as a control. n> 110 cells were analysed for each experimental condition. (O) Time of mitotic exit (NER) as a function of time of entry into mitosis (NEB) in control (DMSO) and SAC inhibitor treated cells in the presence or absence of Wee1 inhibitor. n> 110 cells were analysed for each experimental condition. (P) Duration of mitosis as a function of mitotic entry (NEB) in control (DMSO) and SAC inhibitor treated cells in the presence or absence of Wee1 inhibitor. n> 110 cells were analysed for each experimental condition. Trend lines are shown. (dark blue line: m = 0.00055, R2 = 0.0001, Pearson correlation coefficient, r = 0.0069; red line: m = 0.45, R2 = 0.27, Pearson correlation coefficient, r = 0.44; light blue line: m =

0.00021, R2 = 0.00001, Pearson correlation coefficient, r = 0.0026; yellow line: m = 0.074, R2 = 0.0056, Pearson correlation coefficient, r = 0.0001).

Supplemental Figure S7 (related to Figure 7). ODE model predicts that perturbing positive feedback and not SAC makes duration of mitosis more variable and insulated from upstream cell cycle events. (A-C) Histograms showing duration of mitosis in MCF10A, RPE and HeLa cells, respectively. Cells were either treated with DMSO (control) or Wee1 inhibitor or treated with SAC inhibitor in the presence (Wee1 inh) or absence (DMSO) of Wee1 inhibitor. Mean absolute deviations (MAD) are shown. n=100 cells per experimental condition. (D) Steady state and (E) time activation dynamics of Cdk1-cyclin B1 with changing Wee1 strength. Steady-state solutions of (Cyc B-Cdk1 activation as a function of Cyclin B1 accumulation) of Eqs. (1)-(4), for varying scaling of a_{wee} and b_{wee}, i.e. scaled by a factor 1.5; 1; 0.5; 0.25 (top to bottom). (E) Shows the corresponding time evolution when initialized at [Cyc B] = 10 and [Cyc B-Cdk1] = 5. (F) Steady-state activation dynamics of APC with changing MAD2 strength. Steady-state solutions (APC activity as a function of Cdk1 activity) of Eq. (6), for varying values of b_P = 0.5; 1; 1.5; 2min^{-1}. Red line indicates inactive APC and blue line indicates active APC. (G) Noise distribution functions of cyclin accumulation rate k_S. (H) Noise distribution functions of Mad2 strength b_P.

Supplemental experimental procedures

Cell lines

All the experiments in this study were performed in human MCF10A, RPE and HeLa cell lines. Maintenance MCF10A (ATCC) were cultured in DMEM/F12 (1:1) (Gibco) supplemented with 5% horse serum (LifeTechnologies), EGF (20ng/mL) (PeproTech EC Ltd), Hydrocortizone (0.5mg/mL) (Sigma), Cholera Toxin (100ng/mL) (Sigma), Insulin (10µg/mL) (Sigma), penicillin (100U/mL), streptomycin (100µg/mL) and glutamine (4mM) (Invitrogen). ARPE-19 and RPE-HPV (ATCC) were culture in DMEM/F12 (1:1) (Gibco) supplemented with

10% fetal bovine serum (Gibco), penicillin (100U/mL), streptomycin (100μg/mL) and glutamine (4mM) (Invitrogen). Hela cells (ATCC) were cultured in DMEM (Invitrogen) supplemented with 10% fetal bovine serum (Gibco), penicillin (100U/mL), streptomycin (100μg/mL) and glutamine (4mM) (Invitrogen). MCF7 (ATCC) were cultured in MEM (Gibco) supplemented with 10% fetal bovine serum (Gibco), 1% non-essential amino acids (Gibco), 1% sodium pyruvate (Gibco), penicillin (100U/mL), streptomycin (100μg/mL) and glutamine (4mM) (Invitrogen). Human (H1) embryonic stem cells were cultured in matrigel coated plates and mTESR1 (StemCell Technologies). Mouse (R1) embryonic stem cells were cultured in Knockout™ MEM (Life Technologies), 10% FBS (Gibco), 2-Mercaptoethanol (50μM), MEM non-essential amino acids and LIF recombinant mouse protein (10ng/ml). All cell lines were culture at 37°C, 5% CO_2.

Microscopy

Live cell imaging was performed on either ScanR, a fully motorized and automated inverted epifluorescence microscope system IX83 (Olympus) combined with CellVivo (Olympus) or IncuCyte Zoom® (Essen BioScience). Both equipped with temperature, humidity and CO_2 levels control to keep the sample integrity and perfect focus. ScanR images were typically acquired with a 20x plan (UCPLFLN) fluorescence objective (NA 0.7) and a sCMOS (Orca Flash 4.0, Hamamatsu) camera. LED-based illumination (SpectraX LED, Lumenco) was used for excitation. Excitation (ex) and emission (em) filters were as follows: DAPI ex: 391/20nm, em: 440/521/607/700nm; CFP ex: 438/24nm, em: 460-510nm, GFP/Alexa-488 ex: 474/27nm, em: 440/521/607/700nm; YFP ex: 509/22nm, em: 515-560nm, mCherry ex: 554/23nm, em: 440/521/607/700nm and Alexa-647 ex: 650/13nm, em: 690/50nm. IncuCyte Zoom images were acquired with a 20x plan fluorescence objectives and a CCD camera. Fluorescence excitation (ex) and emission (em) filters were as follows: Green channel ex: 440-480nm, em: 504-544nm; Red channel ex: 565-605nm em: 625-705nm.

A typical experiment to follow cell cycle dynamics would monitor cells from 48 to 72 hours with images taken every 10 minutes. Temperature experiments were done with cells at 37°C for 16 hours after which temperature was shifted to either 34°C or 40°C for further 48h. To test the effect of frame frequency in measurements of mitosis length images were taken every 1, 2, 5 and 10 minutes during 24h. Experiments where Wee1/Myt1 inhibitor was used, cells were monitored for 5 hours, and images were taken every 3-5 minutes. For experiments were other Wee1/Myt1 inhibitor was combined with other inhibitors, cells were incubated for 30 minutes with inhibitors at indicated concentrations before Wee1/Myt1 inhibitor was added.

Mathematical modelling

Various models have been constructed to gain insights into the behavior of the cell cycle. These range from more complicated models involving many explicit molecular reactions (and corresponding ODE equations) (Novak and Tyson, 1993), (Novak, 1993), (Pomerening et al., 2003), (Novak, 2004), (Csikasz-Nagy et al., 2006) to more simple models using only a handful of ODE equations (Yang and Ferrell, 2013), (Gerard et al., 2013), (Tsai et al., 2014), (Gelens et al., 2015). In this work, we take the second approach, motivated by the fact that simple models can often still capture similar dynamical behavior as the more complicated models. Moreover, they have the benefit of containing fewer parameters thereby facilitating parameter selection and gaining more insight into how each individual parameter influences the behavior of the system.

Initially, we constructed a simple model that takes into account the synthesis and destruction of Cyclin B1 and the activation and inactivation of Cdk1-cyclin B1 complexes. Cyclin B1 was assumed to be synthesized and degraded at a constant rates k_s and a_{deg}, respectively. Moreover, we assumed that it bound quickly to Cdk1 and that the Cdk1-cyclin B1 complexes were quickly phosphorylated by the Cdk-activating kinase (CAK). Under these assumptions synthesized Cyclin B1 immediately produces active Cdk1-cyclin B1 complexes. However, it is known that the activity of Cyclin B-Cdk1 is regulated by various other (de)phosphorylations. It is only fully active when Threonine (Thr) 161 is

phosphorylated (by CAK) and Thr 14 and Tyrosine (Tyr) 15 dephosphorylated. The kinase Wee1 (and Myt1) phosphorylates Thr 14 and Tyr 15 and thereby inactivates Cdk1 with a rate constant k_{wee}. Cdk1 itself also inactivates Wee1, leading to a double negative feedback loop (McGowan and Russell, 1993), (Mueller et al., 1995), (Mueller et al., 1995), (Parker and Piwnica-Worms, 1992), (Tang et al., 1993). Similarly, the phosphatase Cdc25 dephosphorylates Tyr15 and activates Cdk1 with a rate constant k_{cdc}, and Cdc25 is in turn activated by Cdk1 forming a positive feedback loop (Solomon et al., 1990), (Hoffmann et al., 1993). These interactions lead to two simple ODE equations describing the time evolution of active Cdk1-cyclin B1 complexes ([Cyc B-Cdk1]) and of total Cyclin B1 ([CycB]):

$$\frac{\mathrm{d[CycB - Cdk1](t)}}{\mathrm{dt}} = k_s - a_{deg}[CycB - Cdk1](t) + k_{cdc}\big([CycB - Cdk1](t)\big)\big([CycB](t) - [CycB - Cdk1](t)\big) - k\_wee\ ([CycB - Cdk1](t))[CycB - Cdk1](t) \qquad (1)$$

$$\frac{d[CycB](t)}{dt} = \mathrm{k_s} - a_{deg}\mathrm{[CycB](t)} \qquad (2)$$

where the rate constants k_{cdc} and k_{wee} depend on the concentration of active Cdk1-cyclin B1 complexes ([Cyc B-Cdk1]). We implemented this dependency by the following two Hill functions with high enough Hill exponents, which is a good approximation as long as these (de)phosphorylations occur on a faster time scale than the modeled time evolution of [Cyc B-Cdk1] and [Cyc B]:

$$k_{cdc}([CycB - Cdk1]) = a_{cdc} + b_{cdc}\frac{\mathrm{[CycB{-}Cdk1]^{n_{cdc}}}}{\mathrm{EC50_{cdc}^{n_{cdc}} + [CycB{-}Cdk1]^{n_{cdc}}}} \qquad (3)$$

$$k_{wee}([CycB - Cdk1]) = a_{wee} + b_{wee}\frac{EC50_{wee}^{n_{wee}}}{EC50_{wee}^{n_{wee}} + [CycB{-}Cdk1]^{n_{wee}}} \qquad (4)$$

This simple ODE system, including the feedback loops involving Wee1 and Cdc25 can turn the system into a bistable switch, as long as Wee1 activity is strong enough compared to Cdc25. This is illustrated in Supplemental Figure S7 (D) where the Cdk1 activity ([Cyc B-Cdk1]) is shown in function of Cyclin B1 levels

([Cyc B]), assuming that there is no synthesis or degradation of Cyclin B1. The parameters corresponding to the Cdc25 and Wee1 feedback are chosen as follows:

$$
\begin{aligned}
a_{cdc} &= 0.5min^{-1} \\
b_{cdc} &= 1.5min^{-1} \\
EC50_{cdc} &= 30nM \\
n_{cdc} &= 10 \\
a_{wee} &= 0.5min^{-1} \\
b_{wee} &= 1min^{-1} \\
EC50_{wee} &= 30nM \\
n_{wee} &= 10 \qquad (5)
\end{aligned}
$$

and the strength of the Wee1 loop (a_{wee} and b_{wee}) was scaled by a factor 1.5, 1, 0.5, 0.25 from top to bottom as shown in Supplementary Figure S7 (D). It is worth noting that while increasing the strength of the Wee1 loop (with respect to the strength of the Cdc25 loop) tends to increase the region of bistability (gray region), decreasing its strength decreases this region of bistability. In addition, when the Wee1 feedback loops are strong, Cdk1 activity can be maintained in a more inactive state for a wider range of Cyclin levels. In contrast, when the Wee1 loops are not able to inhibit Cdk1 activity much, the response curve becomes more linear, such that any increase in Cyclin B1 leads to a proportional increase in Cdk1 activity.

Supplementary Figure S7 (E) shows the system behavior in the presence of constant Cyclin synthesis (k_s = 0.1nMmin^{-1}) and degradation (a_{deg} = 7.5*10^{-4} min^{-1}). The system is initialized at low concentrations of Cyclin B1 ([Cyc B] = 10) and active Cdk1 ([Cyc B-Cdk1] = 5), shown by point (i). Then Cyclin levels ramp up to a final stationary value of [CycB] = $k_s/a_{deg} \approx$ 133nM, point (f) outside of the plotted range. Cdk1 activity behaves dramatically differently in the presence of the Wee1 feedback loops of varying strength. The stronger Wee1 activity, the longer Cdk1 activity is kept low, and the higher and the more abrupt the jump in Cdk1 activity is when Cyclin B1 levels reach the threshold T. This bistability involving Wee1 has been shown to explain the abrupt and all-or-none activation

of Cdk1, and therefore mitotic entry, in various systems (Novak and Tyson, 1993), (Novak, 1993), (Pomerening et al., 2003), (Novak, 2004), (Csikasz-Nagy et al., 2006), (Yang and Ferrell, 2013), (Tuck et al., 2013), (Tsai et al., 2014), (Gelens et al., 2015).

Next, we wanted to include activation of the Anaphase Promoting Complex/ Cyclosome (APC-cdc20) in response to Cdk1 activation. Here, we envisioned that a similar double-negative feedback loop might be present between APC-cdc20 and Mad2 (mitotic arrest deficient 2). Mad2 is a critical component of the mitotic checkpoint complex (MCC) or spindle assembly checkpoint (SAC), which inhibits APC-cdc20 activity. Active Mad2 sequesters Cdc20, an essential APC activator and thereby competes with and antagonizes with APC activation (Foster and Morgan, 2012), (Izawa and Pines, 2012). Furthermore, it has been shown that APC-cdc20 itself also inhibits SAC (which Mad2 is part of), closing the double negative loop (Reddy et al., 2007), (Nilsson et al., 2008), (Izawa and Pines, 2012), (Foster and Morgan, 2012). We implemented such an interaction by assuming that Cdk1 activates the APC-cdc20 in an ultrasensitive manner, as has been shown before (Yang and Ferrell, 2013), (Tsai et al., 2014).

Furthermore, we assumed that Mad2 could inhibit the APC-cdc20 until it reaches a critical level after which Mad2 can no longer effectively inhibit APC-cdc20. The ODE equation describing such interaction between the APC-cdc20 and Mad2 is the following:

$$\frac{d[APC](t)}{dt} = \left(a_K + b_K \frac{[CycB-Cdk1](t)^{n_K}}{[CycB-Cdk1](t)^{n_K} + EC50_K^{n_K}}\right)(1-[APC](t)) - \left(a_P + b_P \frac{EC50_P^{n_P}}{[APC](t)^{n_P} + EC50_P^{n_P}}\right)[APC](t) \quad (6)$$

where the parameters have been chosen as follows:

$$a_K = 0 min^{-1}$$
$$b_K = 0.25 min^{-1}$$
$$EC50_K = 0.18$$
$$n_K = 5$$
$$a_P = 0.025 min^{-1}$$

$$b_P = 0.5min^{-1}$$
$$EC50_P = 0.18$$
$$n_P = 5 \quad (7)$$

Supplemental Figure S7 (F) shows the resulting steady-state solutions (APC-cdc20 activities in function of Cdk1 activity) of Eq. (6), for varying values of b_P = 0.5, 1, 1.5, 2min^{-1}, which effectively increases the strength of APC inhibition by Mad2. One can immediately notice that this single ODE equation also allows for bistability. The width of the bistable region increases with increasing strength of Mad2. The idea of implementing such a additional ODE equation to describe APC activity is that such bistability allows for two things that we believe are important in cell cycle regulation: (i) when increasing Cdk1 activity across the threshold, APC activity greatly increases in a sharp and irreversible manner, and (ii) changing the strength of Mad2 allows to tune this threshold in such a way that when Mad2 activity is strong enough, the APC can never be activated. This way the system remains in the low APC activity state (red) and no metaphase to anaphase transition takes place. In other words, implementing a tunable bistable switch in the activity of APC allows to dynamically regulate (activate and deactivate) the Spindle Assembly Checkpoint (SAC).

Next, we combined Eqs. (1)-(4) with Eq. (6) to turn this into a model for cell cycle oscillations. Once the APC is active, it ubiquitinates Cyclin B1, thus targeting it for degradation by the proteasome, which in turn leads to the inactivation of Cdk1 (Holloway et al., 1993), (King et al., 1995). We assumed that both these events occurred with the same degradation rate b_{deg}, and we implemented additional ultrasensitivity in APC activation to avoid the system getting arrested in interphase, instead of producing regular time-periodic oscillations in Cyclin B1 and Cdk1 activity.

The complete set of equations reads:

$$\frac{d[CycB - Cdk1](t)}{dt} = k_s - a_{deg}[CycB - Cdk1](t) - b_{deg}[APC]^*(t)[CycB - Cdk1](t) + k_{cdc}\big([CycB - Cdk1](t)\big)\big([CycB](t) - [CycB - Cdk1](t)\big) - k_{wee}\big([CycB - Cdk1](t)\big)[CycB - Cdk1](t) \quad (8)$$

$$\frac{d[CycB](t)}{dt} = k_s - a_{deg}[CycB](t) - b_{deg}[APC]^*(t)[CycB](t) \quad (9)$$

$$\frac{d[APC](t)}{dt} = \left(a_K + b_K \frac{[CycB-Cdk1](t)^{n_K}}{[CycB-Cdk1](t)^{n_K} + EC50_K^{n_K}}\right)\big(1 - [APC](t)\big) - (a_P + b_P \frac{EC50_P^{n_P}}{[APC](t)^{n_P} + EC50_P^{n_P}})[APC](t) \quad (10)$$

with $[APC]^*(t) = \frac{[APC](t)^{n_{apc}}}{EC50_{apc}^{n_{apc}} + [APC](t)^{n_{apc}}}$, and the basic parameter set given by:

$$k_s = 0.1 nM min^{-1}$$

$$a_{deg} = 0.001 min^{-1}$$

$$b_{deg} = 0.02 min^{-1}$$

$$a_{cdc} = 0.5 min^{-1}$$

$$b_{cdc} = 1.5 min^{-1}$$

$$EC50_{cdc} = 30 nM$$

$$n_{cdc} = 10$$

$$a_{wee} = 0.5 min^{-1}$$

$$b_{wee} = 1 min^{-1}$$

$$EC50_{wee} = 30 nM$$

$$n_{wee} = 10$$

$$a_K = 0 min^{-1}$$

$$b_K = 0.25 min^{-1}$$

$$EC50_K = 0.18$$

$n_K = 5$

$a_P = 0.025 min^{-1}$

$b_P = 0.5 min^{-1}$

$EC50_P = 0.18$

$n_P = 5$

$EC50_{apc} = 0.5$

$n_{apc} = 10$

Using this set of parameters, Figure 7B-D in the main text shows the resulting cell cycle oscillations. We chose the parameters k_s and b_{deg} such that durations of interphase and mitosis (M-Phase) were realistic. a_{deg} was then chosen to be small enough such that Cyclin B1 accumulates to high enough values to flip the Cdk1-cyclin B1 switch and activate Cdk1. Most of the other parameters are flexible and were chosen to set the threshold values of both the Cdk1-cyclin B1 (Wee1) switch and the Cdk1/APC (Mad2) switch.

Finally, we introduced noise in the model to verify whether this simple model showed qualitatively all properties observed in our experiments, namely that mitosis phase is short, constant, and uncorrelated with the total cell cycle length (which is largely determined by interphase duration). Noise was implemented in the form of random variation (normally distributed) around a mean value corresponding to the standard parameters mentioned before. We therefore added noise with a standard deviation of 0.01nMmin^{-1} to the Cyclin B1 accumulation rate k_s and with a standard deviation of 0.025min^{-1} to the Mad2 strength b_P. This noise introduces changes in cell cycle length. Additionally, in 2% of the simulated cells, we increased the mean value of b_P to 2, mimicking events where the SAC is activated and the cell cycle arrests in M-phase. The resulting distributions of parameter values are shown in Supplemental Figure S7 (G, H).

Using an improved Euler method we then simulated Eqs. (8)-(10) for 150 hours after an initial transient time of 10 hours. We determined whether there

were regular cell cycle oscillations by calculating the oscillation period and verifying whether the system had oscillated with the same period and amplitude for at least three times. If so, we calculated the time in interphase as the time within one oscillation that the system had a Cdk1 activity smaller than 27, while the time in M phase was scored as the time Cdk1 activity was larger than 27. The value 27 was chosen to fall within the range of Cdk1 activities of the middle branch solution in Supplemental Figure S7D-E for all Wee1 strengths. We repeated this simulation 1000 times and then used this data to plot probability density functions of interphase and mitotic durations, as shown in Figure 7E, in the main text. Here, the top panel shows that in the presence of noise, interphase duration greatly varies, while M phase duration stays short and constant. Moreover, in panel G of the same figure in the main text (Figure 7G), the top panel shows that M phase duration was uncorrelated with interphase duration.

Finally, we set out to verify the effect of perturbing one or both of the two bistable switches: the Cdk1-cyclin B1:Wee1 switch and the APC-cdc20:Mad2 switch. We did this by inhibiting Wee1 and Mad2 activity. In the first case (Cdk1-cyclin B1:Wee1 switch), we decreased the rates a_{wee} and b_{wee} by a factor 7. In the second case (APC-cdc20:Mad2 switch), we decreased the rate b_P by a factor 7. The results are shown in the panels Figure 7E and 7G of the main text, and agree qualitatively very well with experiments (Figure 7F, Figure 4F-G, Figure 6C-D and Figure 6G-H).

Supplemental references

Csikasz-Nagy, A., Battogtokh, D., Chen, K.C., Novak, B., and Tyson, J.J. (2006). Analysis of a generic model of eukaryotic cell-cycle regulation. Biophys J *90*, 4361-4379.

Foster, S.A., and Morgan, D.O. (2012). The APC/C subunit Mnd2/Apc15 promotes Cdc20 autoubiquitination and spindle assembly checkpoint inactivation. Molecular cell *47*, 921-932.

Gelens, L., Huang, K.C., and Ferrell, J.E., Jr. (2015). How Does the Xenopus laevis Embryonic Cell Cycle Avoid Spatial Chaos? Cell Rep *12*, 892-900.

Gerard, C., Tyson, J.J., and Novak, B. (2013). Minimal models for cell-cycle control based on competitive inhibition and multisite phosphorylations of Cdk substrates. Biophys J *104*, 1367-1379.

Hoffmann, I., Clarke, P.R., Marcote, M.J., Karsenti, E., and Draetta, G. (1993). Phosphorylation and activation of human cdc25-C by cdc2--cyclin B and its involvement in the self-amplification of MPF at mitosis. The EMBO journal *12*, 53-63.

Holloway, S.L., Glotzer, M., King, R.W., and Murray, A.W. (1993). Anaphase is initiated by proteolysis rather than by the inactivation of maturation-promoting factor. Cell *73*, 1393-1402.

Izawa, D., and Pines, J. (2012). Mad2 and the APC/C compete for the same site on Cdc20 to ensure proper chromosome segregation. The Journal of cell biology *199*, 27-37.

King, R.W., Peters, J.M., Tugendreich, S., Rolfe, M., Hieter, P., and Kirschner, M.W. (1995). A 20S complex containing CDC27 and CDC16 catalyzes the mitosis-specific conjugation of ubiquitin to cyclin B. Cell *81*, 279-288.

McGowan, C.H., and Russell, P. (1993). Human Wee1 kinase inhibits cell division by phosphorylating p34cdc2 exclusively on Tyr15. The EMBO journal *12*, 75-85.

Mueller, P.R., Coleman, T.R., and Dunphy, W.G. (1995). Cell cycle regulation of a Xenopus Wee1-like kinase. Mol Biol Cell *6*, 119-134.

Nilsson, J., Yekezare, M., Minshull, J., and Pines, J. (2008). The APC/C maintains the spindle assembly checkpoint by targeting Cdc20 for destruction. Nature cell biology *10*, 1411-1420.

Novak, B., Tyson J. J. (1993). Modeling the cell division cycle: M-phase trigger, oscillations and size control. Journal of Theorectical Biology *165*, 101-134.

Novak, B., Tyson J. J. (2004). A model for restriction point control of the mammalian cell cycle. Journal of Theorectical Biology *230*, 563-579.

Novak, B., and Tyson, J.J. (1993). Numerical analysis of a comprehensive model of M-phase control in Xenopus oocyte extracts and intact embryos. J Cell Sci *106 (Pt 4)*, 1153-1168.

Parker, L.L., and Piwnica-Worms, H. (1992). Inactivation of the p34cdc2-cyclin B complex by the human WEE1 tyrosine kinase. Science *257*, 1955-1957.

Pomerening, J.R., Sontag, E.D., and Ferrell, J.E., Jr. (2003). Building a cell cycle oscillator: hysteresis and bistability in the activation of Cdc2. Nature cell biology *5*, 346-351.

Reddy, S.K., Rape, M., Margansky, W.A., and Kirschner, M.W. (2007). Ubiquitination by the anaphase-promoting complex drives spindle checkpoint inactivation. Nature *446*, 921-925.

Solomon, M.J., Glotzer, M., Lee, T.H., Philippe, M., and Kirschner, M.W. (1990). Cyclin activation of p34cdc2. Cell *63*, 1013-1024.

Tang, Z., Coleman, T.R., and Dunphy, W.G. (1993). Two distinct mechanisms for negative regulation of the Wee1 protein kinase. The EMBO journal *12*, 3427-3436.

Tsai, T.Y., Theriot, J.A., and Ferrell, J.E., Jr. (2014). Changes in oscillatory dynamics in the cell cycle of early Xenopus laevis embryos. PLoS biology *12*, e1001788.

Tuck, C., Zhang, T., Potapova, T., Malumbres, M., and Novak, B. (2013). Robust mitotic entry is ensured by a latching switch. Biol Open *2*, 924-931.

Yang, Q., and Ferrell, J.E., Jr. (2013). The Cdk1-APC/C cell cycle oscillator circuit functions as a time-delayed, ultrasensitive switch. Nature cell biology *15*, 519-525.
